# Tubular epithelial cell-derived extracellular vesicles carrying serum amyloid A1 exacerbate sepsis-associated acute kidney injury by promoting NETs formation

**DOI:** 10.3389/fimmu.2025.1654295

**Published:** 2025-08-27

**Authors:** Yang Jiao, Mei Liu, Xin Xie, Mengying Pi, Luyang Zhou, Wei Zhu, Jia Song, Ti Zhang, Zhengliang Ma, Xiaoping Gu

**Affiliations:** ^1^ Department of Anesthesiology, Nanjing Drum Tower Hospital, The Affiliated Hospital of Nanjing University Medical School, Nanjing, China; ^2^ National Clinical Research Center of Kidney Diseases, Jinling Hospital, Nanjing University School of Medicine, Nanjing, China

**Keywords:** sepsis-associated acute kidney injury, tubular epithelial cells, extracellular vesicles, neutrophil extracellular traps, serum amyloid A1

## Abstract

**Introduction:**

Sepsis-associated acute kidney injury (SA-AKI) is a highly lethal condition with a rapid onset, and effective treatments are lacking because the molecular pathogenesis remains unclear. Tubular epithelial cells (TECs) have increasingly been recognized as driving forces in the progression of kidney diseases, partly through the release of extracellular vesicles (EVs) carrying proinflammatory cargos. However, the role of TEC-derived EVs on neutrophil extracellular traps (NETs) formation, which is an established feature of sepsis, and SA-AKI remains unclear.

**Methods:**

EVs isolated from phosphate buffer saline (PBS)/lipopolysaccharide (LPS)-treated TECs were injected intravenously into C57BL/6J wild type mice to determine whether TECs-derived EVs can directly induce NETs formation and kidney injury. Proteomics and single-cell RNA sequencing analysis were used to screen the key molecules that mediate the effects of TECs-derived EVs. EVs secretion from TECs and serum amyloid A1 (SAA1) expression in TECs were specifically inhibited via adeno-associated virus (AAVs). Finally, the association between SAA1 level in plasma EVs and clinical features of septic patients was determined.

**Results:**

This study demonstrated that EVs secreted from LPS-stimulated TECs exacerbated AKI by promoting NETs formation. Specifically blocking EVs secretion from TECs via AAVs reduced NETs formation and alleviated LPS-induced AKI. Bioinformatics analysis suggested that LPS increased SAA1 expression in TECs, and then released extracellularly through EVs. Further mechanistic studies revealed that SAA1 packaged in TECs-derived EVs was responsible for NETs formation and AKI via activation of the TLR4/p38 MAPK signaling pathway in neutrophils. Specifically inhibiting SAA1 upregulation in TECs via AAVs also reduced NETs formation and alleviated LPS-induced AKI. Interestingly, modulating EVs release from TECs or SAA1 expression in TECs also alleviated remote lung injury induced by LPS, indicated that TECs-derived EVs may participate in kidney‒lung crosstalk during sepsis. Furthermore, plasma TECs-derived EVs proportion and SAA1 expression in plasma EVs may be promising prognostic indexes for SA-AKI patients.

**Discussion:**

Here, we explored a new mode of TECs-neutrophils crosstalk mediated by EVs during SA-AKI, and strategies to modify TECs-derived EVs and the cargo SAA1 could be a new avenue for developing therapeutics against SA-AKI.

## Introduction

Sepsis—a life-threatening global health crisis—is a leading cause of hospital mortality. It arises from infection-induced immune hyperactivation and cytokine storms, resulting in tissue damage and multi-organ dysfunction, including acute kidney injury (AKI). Sepsis-associated acute kidney injury (SA-AKI) affects nearly 60% of septic patients, significantly compounding morbidity and mortality risks. However, the pathophysiology of SA-AKI remains incompletely understood (1).

Neutrophils are important innate immune cells playing crucial roles in the pathogenesis of sepsis (2). Neutrophils activated locally execute pathogen defense through oxidative burst, phagocytosis, and NETs formation (3). Composed of DNA, histones and granule proteins, NETs in sepsis function dually: constraining pathogens but causing tissue injury when overproduced (4). One preclinical study propose that interrupting NETs formation in combination with monoclonal antibody might be a feasible therapeutic strategy for septic AKI (5). Therefore, reducing NETs formation is crucial for alleviating kidney injury.

Extracellular vesicles (EVs)-mediated intercellular communication critically regulates kidney injury progression (6, 7). Our previous studies have shown that EVs from innate immune cells could modulate NETs formation and thus participate in the pathogenesis of sepsis. EVs-carrying microRNAs and CCL-2 mRNA have the tropism to macrophages and provoke tubulointerstitial inflammation and kidney injury (8–10). However, the interplay among NETs formation, SA-AKI and EVs needs to be further addressed.

SA-AKI is hallmarked by renal inflammation, apoptosis, and oxidative injury (11). Tubular epithelial cells (TECs) are the most populous cell type in the kidney and now recognized not only as injury victims but also as pathogenic mediators accelerating kidney disease progression. Chemokines/cytokines from damaged TECs recruit mainly neutrophils and macrophages to injured kidneys. These infiltrating cells release cytokines that drive further tubular injury, creating a vicious cycle (10). Therefore, we hypothesize that TECs-derived EVs modulate NETs formation and promote SA-AKI progression.

Serum amyloid A (SAA) is a family of acute-phase proteins, the plasma levels of which may increase >1000-fold in acute inflammatory states. SAA1 plus several conventional biomarkers including C-reactive protein, procalcitonin are extensively investigated to diagnose sepsis (12, 13). In addition to be regarded as biomarkers, recent study showed that SAA1 could induce neutrophilic airway inflammation by activating neutrophils along with NETs formation (14). Previous study also suggested that SAA1 was enriched in plasma EVs obtained from septic patients (15). In this study, we aimed to investigate whether TECs-derived EVs could deliver SAA1 to induce NETs formation and SA-AKI progression.

## Materials and methods

### Patient samples and ethics statement

We recruited 26 patients with early (less than 24 h) diagnosis of sepsis who were admitted to the intensive care unit (ICU) of Nanjing Drum Tower Hospital (Nanjing, China) between February and May 2024 (**Supplementary Table S1**). Sepsis was diagnosed according to the Sepsis-3 criteria (16). Acute kidney injury (AKI) was defined using the KDIGO criteria (an increase in serum creatinine (Scr) of ≥0.3 mg/dl within 48 hours or ≥50% from baseline) (17). Pregnant women and patients under 18 years of age and those with existing AKI before admission to the ICU, severe anemia, active bleeding, or chemotherapy were excluded. Healthy age-/sex-matched donors served as controls for septic patients. The study was conducted in accordance with the Declaration of Helsinki, and the protocol was approved by the institutional ethics and review board of Nanjing Drum Tower Hospital (Approval No. 2024-102-02). Informed consent was obtained from the participating volunteers, patients or their representatives.

### Animals

Male C57BL/6J mice (6–8 weeks old, weighting 20–25 g) were purchased from (Vital River Laboratories, Zhejiang, China) were fed under a specific pathogen-free environment in the Laboratory Animal Center of Nanjing First Hospital. All animal experiments were conducted under the rules approved by the Ethics Committee of Nanjing First Hospital (Approval No. DWSY-23011315). The septic AKI mice models were induced by intraperitoneal (*i.p.*) injection of LPS (10 mg/kg of body weight, Sigma-Aldrich, USA) as previously described (18). The control mice were injected with *i.p.* an equal volume of phosphate buffer saline (PBS). To degrade NETs or inhibit EVs release, DNase I (5 mg/kg) or GW4869 (2.5 µg/g) was given with an intraperitoneal injection. To explore EVs function *in vivo*, mice were intravenously (*i.v.*) injected with TECs-derived EVs (300 μg/mouse) using 31-gauge insulin syringes. 24 h later, blood samples were collected for plasma creatinine (Cr), blood urea nitrogen (BUN) and NETs components (MPO-DNA complexes and dsDNA) measurement. Kidney samples were collected for histological analysis and NETs evaluation.

### Knockdown of Rab27a and SAA1 in TECs *in vivo*

To specifically knockdown Rab27a and SAA1 in renal TECs, as previously described (19), we conducted adeno-associated viruses (AAV9) carrying short hairpin RNA against Rab27a/SAA1 and Ksp-cadherin (a unique, tissue-specific member of the cadherin family that is exclusively expressed in renal tubular epithelial cells) which was used as the upstream promoter of Rab27a/SAA1 (GeneChem, Shanghai, China). GFP linked to each shRNA cassette was cloned downstream of the Ksp-cadherin promoter in the AAV9 vector plasmid. Eight-week-old male C57BL/6J mice were administrated 1 × 10^12^ gv/mouse AAVs via tail vein injection one week before LPS injection. The target sequence for Rab27a was 5’-GCTTAACCACTGCATTCTTCA-3’. The target sequences for SAA1 were 5’-GAAGAGAGGCCTTTCAGGAAT-3’, 5’-GGACATGAGGACACCATTGCT-3’, 5’-CTCCTATTAGCTCAGTAGGTT-3’. The insert sequence for negative control (NC) was 5’- TTCTCCGAACGTGTCACGT-3’.

### Cell culture and treatment

The human proximal tubule cell line (HK-2) was cultured in DMEM/F12 medium (Gibco, USA) supplemented with 1% (v/v) penicillin streptomycin (P/S, Gibco, USA) and 10% fetal bovine serum (FBS, Viva cell, China) at 37°C. An immortalized mouse renal tubular epithelial cells (TCMK-1 cells) were maintained in DMEM (Gibco, USA) supplemented with 1% (v/v) P/S (Gibco, USA) and 10% FBS (Viva cell, China) at 37°C. Both HK-2 and TCMK-1 cells were purchased from the American Type Culture Collection. To obtain TECs-derived EVs, HK-2 or TCMK-1 cells were treated with PBS or LPS (1 μg/ml) at 37°C for 24 hours (h) in medium containing 10% exosome-free FBS (#EXO-FBS-50A-1; System Biosciences, Palo Alto, CA, USA), and the supernatant were collected for EVs isolation.

### siRNA and transfection

To delete SAA1 expression in TECs-EVs, HK-2 or TCMK-1 cells were transfected with 50 nM of siRNA against human or mouse SAA1 respectively (RiboBio, Guangzhou, China) using Lipomaster 3000 Transfection Reagent (Vazyme, Nanjing, China) per manufacturer’s recommendations. A scramble sense-control was used as negative control. The target sequences of siRNAs for human SAA1 were as follows: (1) 5’-GATCAGGCTGCCAATGAAT-3’; (2) 5’-GAGAGAATATCCAGAGATT-3’; (3) 5’-GCGATGCCAGAGAGAATAT-3’. The target sequences of siRNAs for mouse SAA1 were as follows: (1) 5’-CUCCUGGACUGCCUGACAA-3’; (2) 5’-GAACUAUGAUGCUGCUCAA-3’; (3) 5’-UGUUCACGAGGCUUUCCAA-3’. After 24 h of siRNA treatment, the culture medium was changed to medium containing 10% exosome-free FBS plus LPS (1 µg/ml) for another 24 h. At the end of the experiments, cells and EVs in the supernatant were collected and the level of protein expression was evaluated by western blotting.

### EVs isolation and characterization

EVs were isolated from the supernatant of HK-2 or TCMK-1 cells treated with PBS (Ctrl-EVs) or LPS (LPS-EVs) using ExoQuick-TC (#EXOTC10A-1; System Biosciences, USA) according to the manufacturer’s instructions. For kidney EVs extraction, we conducted a mouse model of cecal ligation and puncture (CLP) to mimic clinical sepsis as described previously (20). At 24 h after CLP, 100 mg of kidney cortex was collected and submitted to tissue digestion with collagenase and trypsin for 120 minutes at 37°C (10). Then the sample was submitted for EVs extraction using ExoQuick-TC according to the manufacturer’s instructions. Plasma samples from septic patients were collected for EVs purification using differential ultracentrifugation as described previously (21). Plasma was sequentially centrifuged (300 × g/10 min, 2000 × g/20 min, 10,000 × g/30 min; 4°C), filtered (0.22 μm; Merck Millipore SLGP033RB), and ultracentrifuged (100,000 × g/3 h; SW28 Ti rotor). The pellet was washed via PBS resuspension/re-ultracentrifugation (100,000 × g/3 h) and finally resuspended in 200 μl PBS. EVs characterization methods are in **Supplementary Materials**.

### Neutrophil isolation and *in vitro* co–culture experiments

Neutrophils were isolated from healthy donor venous blood using Polymorphprep™ (#1114742, Axis-Shield, Norway) per manufacturer’s protocol. Isolated cells (90% purity, 95% viability by flow cytometry/Trypan blue) were resuspended in complete RPMI 1640 (1% FBS, 50 μg/ml penicillin/streptomycin) at 10^6^ cells/ml.

Freshly isolated neutrophils were co-cultured with Ctrl-EVs or LPS-EVs (100 μg/ml) for 3 h at 37°C. PBS was used as negative control. To inhibit MAPK pathway activation, SB203580 was added to the culture medium at a final concentration of 30 µM about 30 min before co-cultured with EVs. To block formyl peptide receptor 2 (FPR2), Toll-like receptor 2 (TLR2) or TLR4 activation, WRW4 (10 µM), C29 (50 µM) or TAK-242 (50 µM) were added to the culture medium about 30 min before co-cultured with EVs respectively.

### Statistical analysis

Data normality was assessed by Shapiro-Wilk test. Normally distributed data are expressed as mean ± SEM and analyzed by two-tailed Student’s t-test (two groups) or one-way ANOVA with Tukey’s *post hoc* test (multiple groups). Non-normal data are presented as median ± IQR and analyzed by Mann-Whitney U test (two groups) or Kruskal-Wallis with Dunn’s test (multiple groups). Correlations used Spearman’s method. Statistical significance was defined as *P* < 0.05 (GraphPad Prism 8).

More detailed methods are described in **Supplementary Methods**.

## Results

### NETs contributed to kidney injury in an LPS-induced AKI model

**Figure 1:**
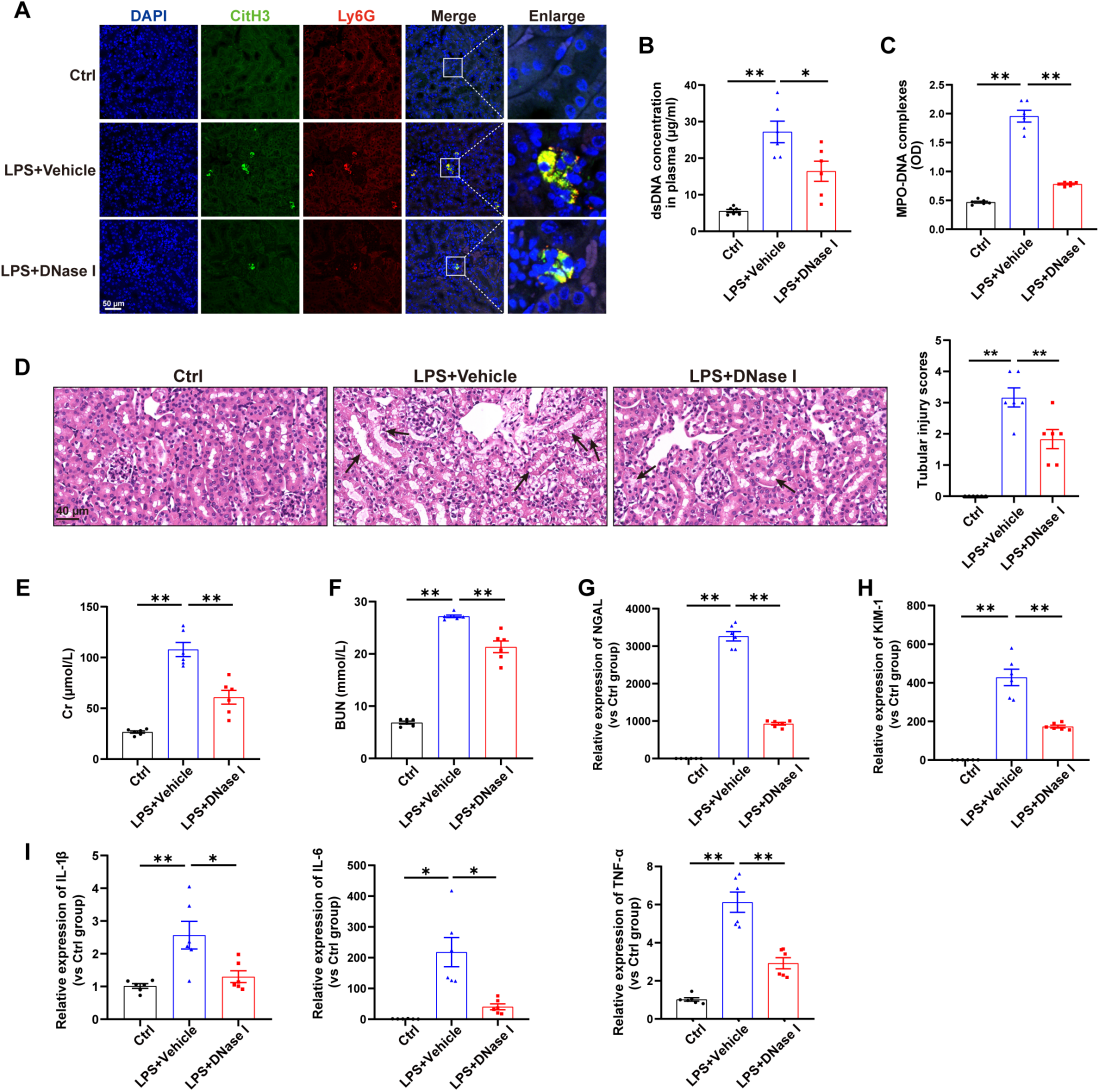
NETs contribute to kidney injury in an LPS-induced AKI model. WT C57BL/6J mice were intraperitoneally injected with LPS (10 mg/kg body weight) for 24 h. The control mice were injected with an equal volume of PBS. DNase I (5 mg/kg) was given via an *i.p.* injection 30 min after LPS, and an equal volume of normal saline was used as the vehicle. **(A)** Representative images showing the presence of NETs (Ly6G, red; citrullinated H3, green) in kidney tissues. Nuclei were counterstained with DAPI (blue). Scale bar, 50 μm. **(B, C)** Quantification of dsDNA and circulating NETs structures (MPO–DNA complexes) in the plasma of mice via PicoGreen fluorescence quantification and ELISA, respectively. **(D)** Representative images of H&E-stained kidneys (original magnification, 400×). Scale bars: 40 μm. The black arrows indicate tubule damage. Quantification of tubular injury via H&E staining. **(E, F)** Quantitation of Cr and BUN in blood samples from mice in each group. **(G-I)** RT–qPCR analysis of NGAL, KIM-1, IL-1β, IL-6 and TNF-α mRNA levels in the kidney. One-way analysis of variance with Tukey’s multiple comparisons test was used for the analysis. The graphs present the means ± SEMs, n = 6; *P < 0.05, **P < 0.01 compared between two groups.

First, a mouse model of sepsis induced by LPS challenge was used to detect the formation of NETs. As shown in **Figure 1A**, immunofluorescence labeling via anti-citrullinated histone H3 (citH3) and anti-Ly6G antibodies revealed increased NETs formation in the kidneys induced by LPS, and the levels of NETs components in the plasma were also significantly increased following LPS challenge (**Figures 1B-C**). For determination of the exact role of NETs in LPS-induced AKI, NETs were degraded by DNase I (a well-established inhibitor of NETs formation) 30 min after LPS administration, and as demonstrated by immunofluorescence, Picogreen and ELISA, NETs formation was significantly decreased (**Figures 1A-C**). Furthermore, H&E staining revealed that DNase I significantly ameliorated the kidney damage induced by LPS (**Figure 1D**) and reduced the plasma Cr and BUN levels (**Figures 1E-F**). qPCR analyses indicated that DNase I treatment attenuated the changes in kidney injury markers (NGAL and KIM-1) and inflammatory factors (IL-1β, IL-6 and TNF-α) caused by LPS challenge (**Figures 1G-I**). Collectively, these findings indicate that LPS stimulates NETs formation to induce kidney injury.

### Inhibition of total EVs release by GW4869 reduced NETs formation and alleviated LPS-induced AKI

**Figure 2:**
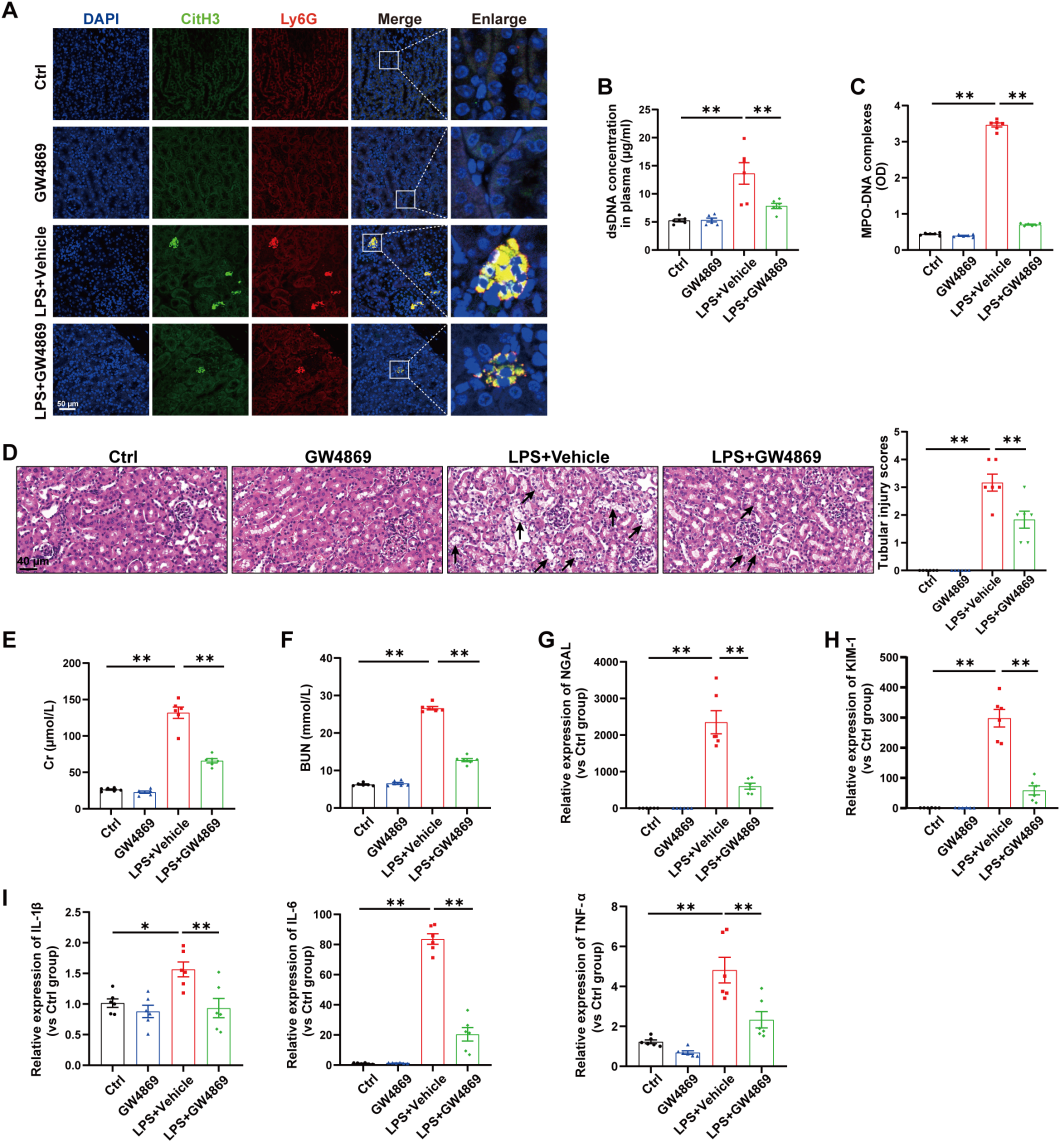
Inhibition of total EVs release by GW4869 reduced NETs formation and alleviated LPS-induced AKI. GW4869 (2.5 µg/g) was given via an *i.p.* injection 1 h before LPS administration, and an equal volume of DMSO was used as the vehicle. **(A)** Representative images showing the presence of NETs (Ly6G, red; citrullinated H3, green) in kidney tissues. Nuclei were counterstained with DAPI (blue). Scale bar, 50 μm. **(B, C)** Quantification of dsDNA and circulating NETs structures (MPO–DNA complexes) in the plasma of mice via PicoGreen fluorescence quantification and ELISA, respectively. **(D)** Representative images of H&E-stained kidneys (original magnification, 400×). Scale bars: 40 μm. The black arrows indicate tubule damage. Quantification of tubular injury via H&E staining. **(E, F)** Quantitation of Cr and BUN in blood samples from mice in each group. **(G-I)** RT–qPCR analysis of NGAL, KIM-1, IL-1β, IL-6 and TNF-α mRNA levels in the kidney. One-way analysis of variance with Tukey’s multiple comparisons test was used for the analysis. The graphs present the means ± SEMs, n = 6; *P < 0.05, **P < 0.01 compared between two groups.

Next, we investigated the relationship between EVs release and NETs formation during SA-AKI. GW4869, a noncompetitive phospholipase inhibitor, was used to block total EVs synthesis/secretion, and NETs formation was substantially inhibited in the kidneys and plasma of septic mice (**Figures 2A-C**). More importantly, the tubular injury scores, plasma Cr and BUN levels, kidney injury markers and inflammatory factors in the kidneys were all reduced by GW4869 (**Figures 2D-I**), indicating that EVs are involved in NETs formation and kidney injury during LPS-induced AKI.

### EVs secreted from LPS-stimulated TECs promoted NETs formation and exacerbated AKI *in vivo*

**Figure 3:**
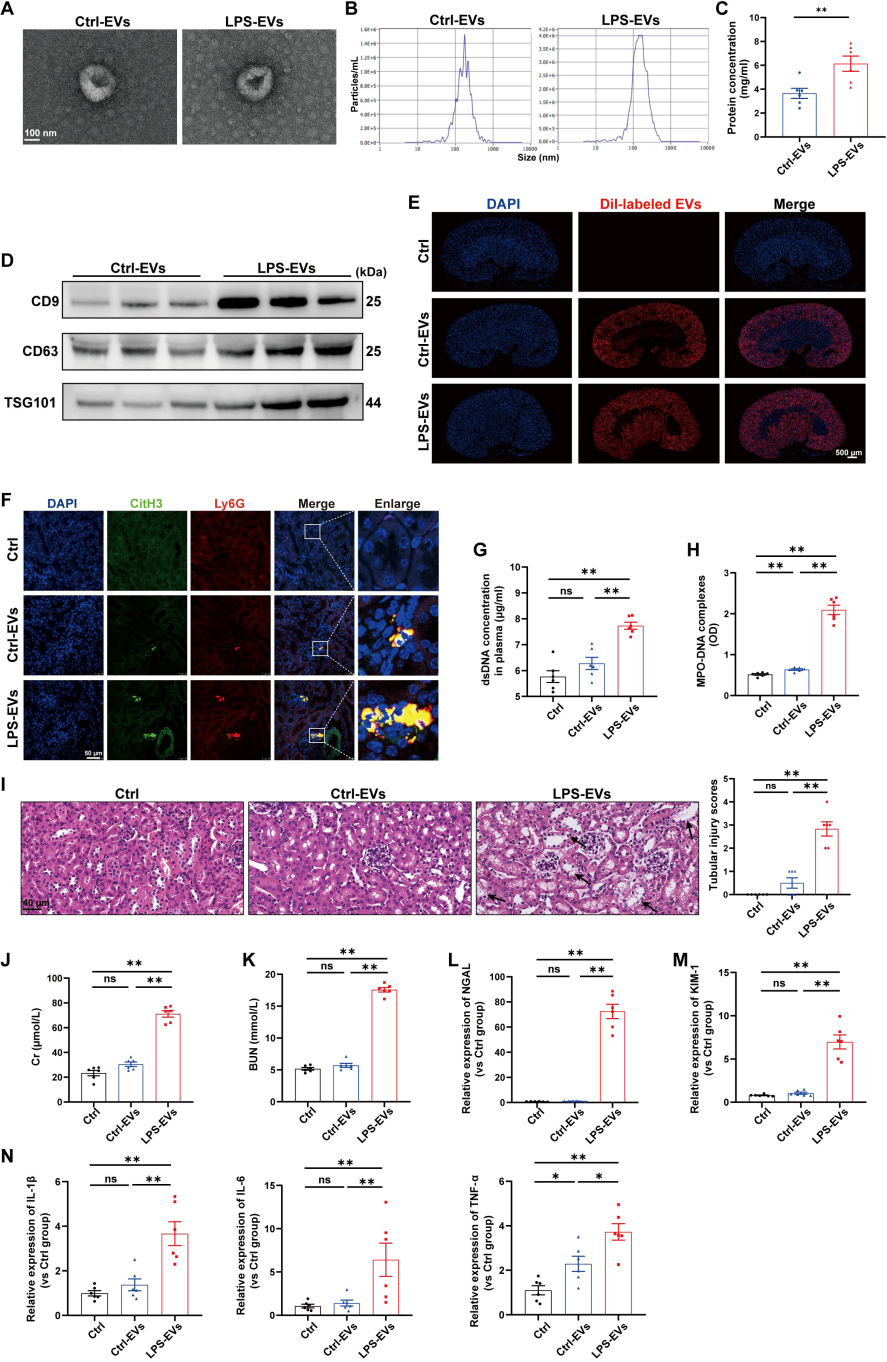
EVs secreted from LPS-stimulated TECs promoted NETs formation and aggravated AKI *in vivo*. **(A)** Electron micrograph of EVs isolated from the supernatant of PBS/LPS (1 µg/ml)-treated TCMK-1 cells (Ctrl-EVs/LPS-EVs). Scale bar, 100 nm. **(B)** EVs size distribution was measured via NanoSight tracking analysis. **(C)** The protein concentration of EVs was determined via a BCA protein assay kit. **(D)** CD9, CD63 and TSG101 protein expression in EVs was quantified by Western blotting with equal amounts of EVs protein (20 μg). **(E–N)** WT C57BL/6J mice were *i.v.* administered PBS (Ctrl group), Ctrl-EVs or LPS-EVs (300 μg/mouse) for 24 h **(E)** Representative images of fluorescent signals in the kidneys of mice *i.v.* injected with Dil-labeled EVs (red). Nuclei were counterstained with DAPI (blue). Scale bar, 500 μm. **(F)** Representative images showing the presence of NETs (Ly6G, red; citrullinated H3, green) in kidney tissues. Nuclei were counterstained with DAPI (blue). Scale bar, 50 μm. **(G, H)** Quantification of dsDNA and MPO-DNA complexes in the plasma of the mice. **(I)** Representative images of H&E-stained kidneys (original magnification, 400×). Scale bars: 40 μm. The black arrows indicate tubule damage. Quantification of tubular injury via H&E staining. **(J, K)** Quantitation of Cr and BUN in blood samples from mice in each group. **(L-N)** RT–qPCR analysis of NGAL, KIM-1, IL-1β, IL-6 and TNF-α mRNA levels in the kidney. Student’s t test **(C)** or one-way analysis of variance with Tukey’s multiple comparisons test **(G–N)** was used for the analysis. The graphs present the means ± SEMs, n = 6; *P < 0.05, **P < 0.01 compared between two groups. ns, not significant.

Previous studies have shown that TECs-derived EVs function as active vectors that play essential roles in the pathogenesis of kidney disease (22). However, whether EVs contribute to the communication between tubules and neutrophils during AKI remains unknown. To address this question, we treated mouse renal TECs (TCMK-1 cells) with LPS (1 µg/ml) to simulate sepsis *in vitro*. After 24 h, EVs in the supernatant were isolated and characterized via transmission electron microscopy, NTA, and western blotting (**Figures 3A-D**). For determination of whether TECs-derived EVs can directly induce NETs formation and kidney injury, TECs-derived EVs were injected *i.v.* into C57BL/6J WT mice, and fluorescence imaging revealed that Dil-labeled EVs accumulated in kidney tissues (**Figure 3E**). Immunofluorescence revealed that LPS-EVs (EVs from LPS-stimulated TCMK-1 cells) promoted NETs formation in kidney tissues (**Figure 3F**) and increased the NETs concentration in the plasma (**Figures 3G-H**). Moreover, more histological lesions were observed in the mouse kidney after the administration of LPS-EVs than after administration of Ctrl-EVs (**Figure 3I**). The plasma Cr and BUN levels, kidney injury markers and inflammatory factors in kidney tissues were significantly increased in the LPS-EVs group (**Figures 3J-N**), suggesting that the kidney injury induced by LPS-EVs occurred in parallel with NETs formation.

To further establish a causal relationship between LPS-EVs-induced NETs formation and kidney injury, we administered DNase I 30 min after LPS-EVs injection to degrade NETs. The results showed that DNase I successfully reduced NETs formation (**Supplementary Figures S1A-C**) and alleviated kidney injury induced by LPS-EVs (**Supplementary Figures S1D-I**). These data indicate that EVs secreted from LPS-stimulated TECs exacerbated AKI by promoting NETs formation.

### EVs secreted from LPS-stimulated TECs promoted NETs formation *in vitro*

**Figure 4:**
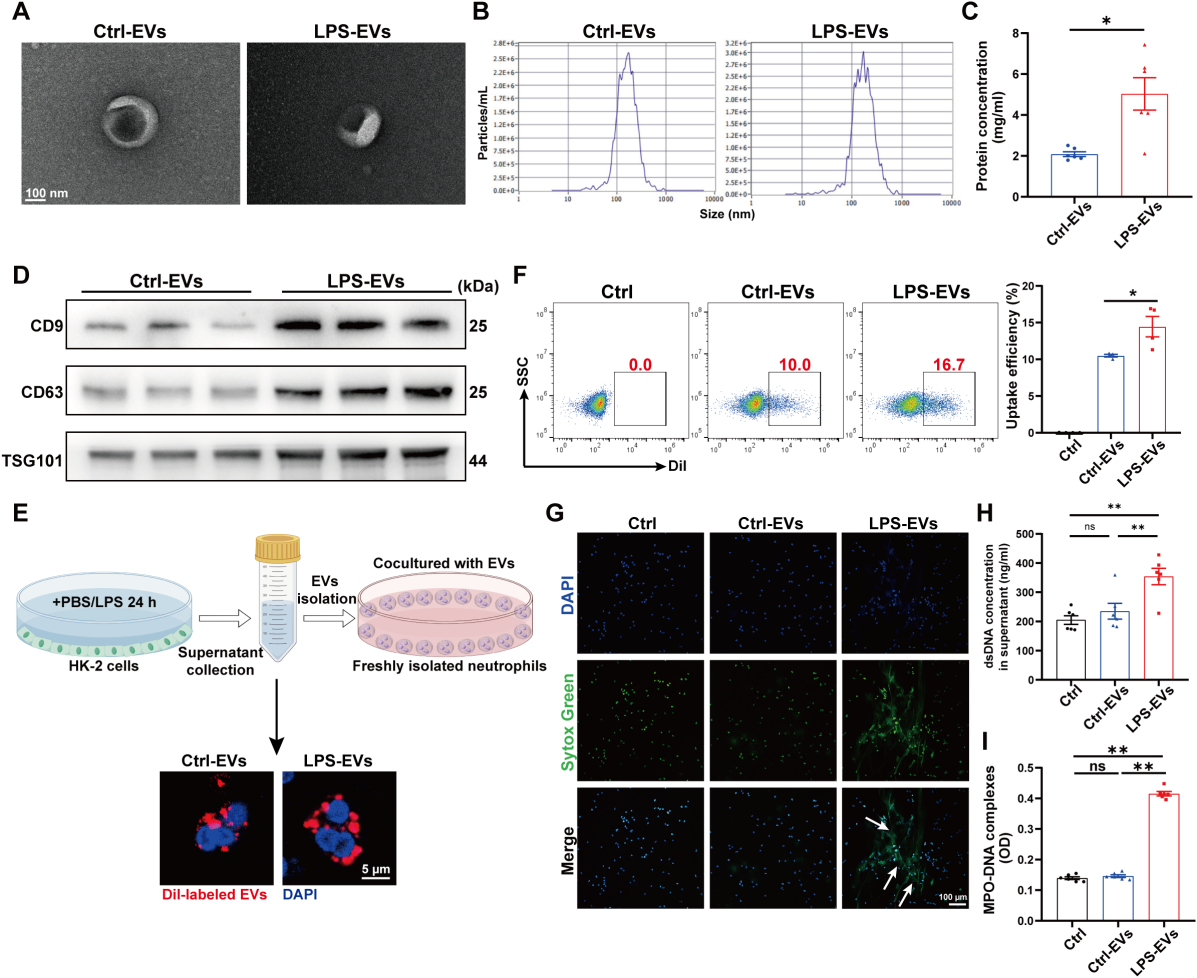
EVs secreted from LPS-stimulated TECs promoted NETs formation *in vitro*. **(A)** Electron micrograph of EVs isolated from the supernatant of PBS/LPS (1 µg/ml)-treated HK-2 cells (Ctrl-EVs/LPS-EVs). Scale bar, 100 nm. **(B)** EVs size distribution was measured via NanoSight tracking analysis. **(C)** The protein concentration of EVs was determined via a BCA protein assay kit. **(D)** CD9, CD63 and TSG101 protein expression in EVs was quantified by Western blotting with equal amounts of EVs protein (20 μg). **(E)** Immunofluorescence images showing neutrophils freshly isolated from healthy volunteers incubated with Dil-labeled EVs (red) for 3 h Nuclei were counterstained with DAPI (blue). Scale bar, 5 μm. **(F)** Uptake efficiency of Dil-labeled EVs by neutrophils was measured by flow cytometry. SSC, side scatter. **(G)** Typical images of NETs formation following 3 h of treatment with Ctrl-EVs/LPS-EVs (100 µg/ml) were obtained via confocal microscopy via Sytox Green (green), where the white arrows indicate NETs. Scale bar, 100 μm. **(H, I)** Quantification of dsDNA and NETs components (MPO–DNA complexes) in the supernatants of cultured neutrophils via the PicoGreen assay and ELISA, respectively. Student’s t test **(C)** or one-way analysis of variance with Tukey’s multiple comparisons test **(F-I)** was used for the analysis. The graphs present the means ± SEMs, n = 4–6; *P < 0.05, **P < 0.01 compared between two groups. ns, not significant.

To further demonstrate the ability of LPS-EVs to stimulate NETs formation, we established an *in vitro* coculture system by coculturing neutrophils freshly isolated from healthy volunteers with TECs-derived EVs. The same species of cell line, the human proximal tubule cell line (HK-2), was used in this part. First, we isolated EVs from HK-2 cells treated with PBS/LPS (1 µg/ml) for 24 h. The isolated EVs were validated via transmission electron microscopy, NTA, and western blotting (**Figures 4A-D**). After incubating neutrophils *in vitro* with Dil-labeled EVs for 3 h, we observed neutrophil internalization of TECs-derived EVs (**Figure 4E**), and the uptake efficacy of LPS-EVs by neutrophils was greater than that of Ctrl-EVs (**Figure 4F**). Confocal microscopy revealed obvious NETs formation following LPS-EVs treatment *in vitro* (**Figure 4G**), and the NETs concentration was significantly increased in the supernatant of the LPS-EVs-treated group (**Figures 4H, I**).

### Blocking EVs secretion from TECs reduced NETs formation and alleviated LPS-induced AKI

**Figure 5:**
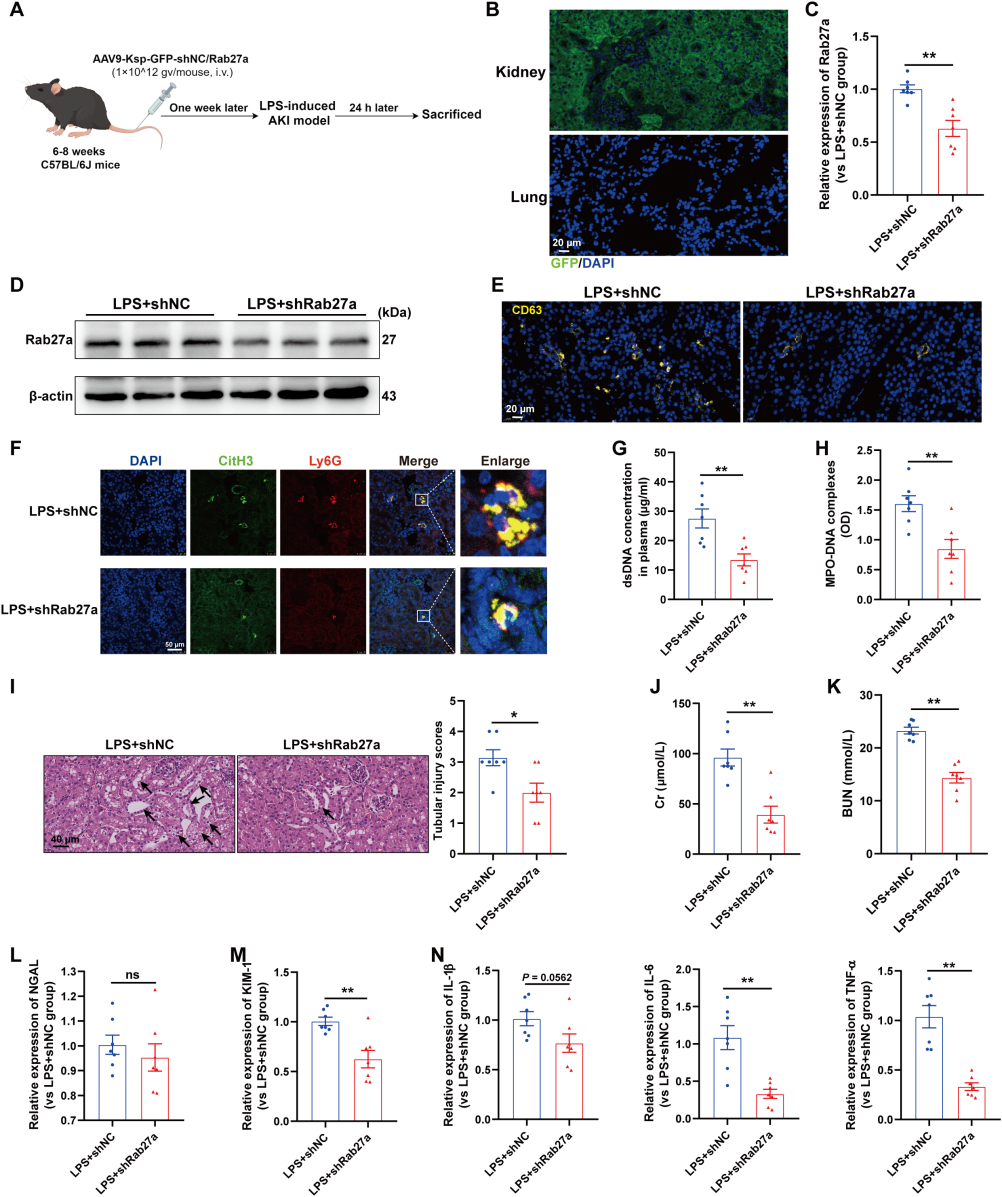
Blocking EVs secretion from TECs reduced NETs formation and alleviated LPS-induced AKI. **(A)** Schematic diagram of the experimental design. Briefly, the mice were subjected to LPS stimulation at 7 days after AAV9-Ksp-GFP-shNC/Rab27a injection and were sacrificed at 24 h after LPS. Schematic was created with Figdraw.com. **(B)** Representative images of GFP fluorescence in the kidney and lung tissues of the AAV9-GFP-injected mice. Scale bar, 20 µm. **(C, D)** Rab27a mRNA and protein expression in kidney tissues was determined after the mice were treated with AAV9-shNC/Rab27a. **(E)** Representative images of staining for the classic EVs marker CD63 in kidneys. Scale bar, 20 µm. **(F)** Representative images showing the presence of NETs (Ly6G, red; citrullinated H3, green) in kidney tissues. Nuclei were counterstained with DAPI (blue). Scale bar, 50 μm. **(G, H)** Quantification of dsDNA and MPO-DNA complexes in the plasma of the mice. **(I)** Representative images of H&E-stained kidneys (original magnification, 400×). Scale bars: 40 μm. The black arrows indicate tubule damage. Quantification of tubular injury via H&E staining. **(J, K)** Quantitation of Cr and BUN in blood samples from mice in each group. **(L-N)** RT–qPCR analysis of NGAL, KIM-1, IL-1β, IL-6 and TNF-α mRNA levels in the kidney. Student’s t test was used for the analysis. The graphs present the means ± SEMs, n = 7; *P < 0.05, **P < 0.01 compared between two groups. ns, not significant.

We subsequently investigated whether impaired EVs secretion from TECs could inhibit NETs formation and then alleviate LPS-induced AKI. Since the small GTPase Rab27a plays a well-established role in EVs release (23), we generated AAV9 carrying shRNAs against Rab27a and Ksp-cadherin (a unique, tissue-specific member of the cadherin family that is exclusively expressed in renal TECs), which was used as the upstream promoter of Rab27a to specifically inhibit EVs secretion from TECs, as previously described (19). As shown in **Figure 5A**, C57BL/6J mice were injected via the tail vein with AAV9-Ksp-GFP-shRab27a (AAV9-shRab27a) or the control vector AAV9-Ksp-GFP-shScramble (AAV9-shNC). One week after injection, these mice were used to establish the LPS-AKI model. GFP fluorescence was clearly observed in nearly 100% of the tubular cells but was not detected in the lung tissue (**Figure 5B**). AAV9-shRab27a significantly decreased the Rab27a mRNA and protein levels in the kidney (**Figures 5C-D**), whereas AAV9-shRab27a did not affect Rab27 mRNA expression in the lung (**Supplementary Figure S2A**), indicating that AAV9-shRab27a-induced Rab27 knockdown was specific to TECs. Further immunofluorescence staining revealed that the expression of the EVs marker CD63 was significantly decreased in the kidneys of the AAV9-shRab27a LPS-AKI mice (**Figure 5E**). In addition, a notable decrease in renal NETs formation and the plasma NETs concentration was detected in the AAV9-shRab27a LPS-AKI group compared with the control group (**Figures 5F-H**). Moreover, histological lesions, plasma Cr and BUN levels, kidney injury markers and inflammatory factors in kidney tissues were decreased in the AAV9-shRab27a LPS-AKI mice (**Figures 5I-N**). Thus, the inhibition of EVs secretion from TECs reduced NETs formation and alleviated LPS-induced AKI.

Interestingly, we also evaluated lung tissues and found that specifically blocking EVs secretion from TECs decreased NETs formation in the lung and alleviated lung injury induced by LPS (**Supplementary Figures S2B-D**), indicating that during sepsis, TECs-derived EVs could mediate remote organ injury.

### SAA1 was enriched in EVs secreted from LPS-stimulated TECs

**Figure 6:**
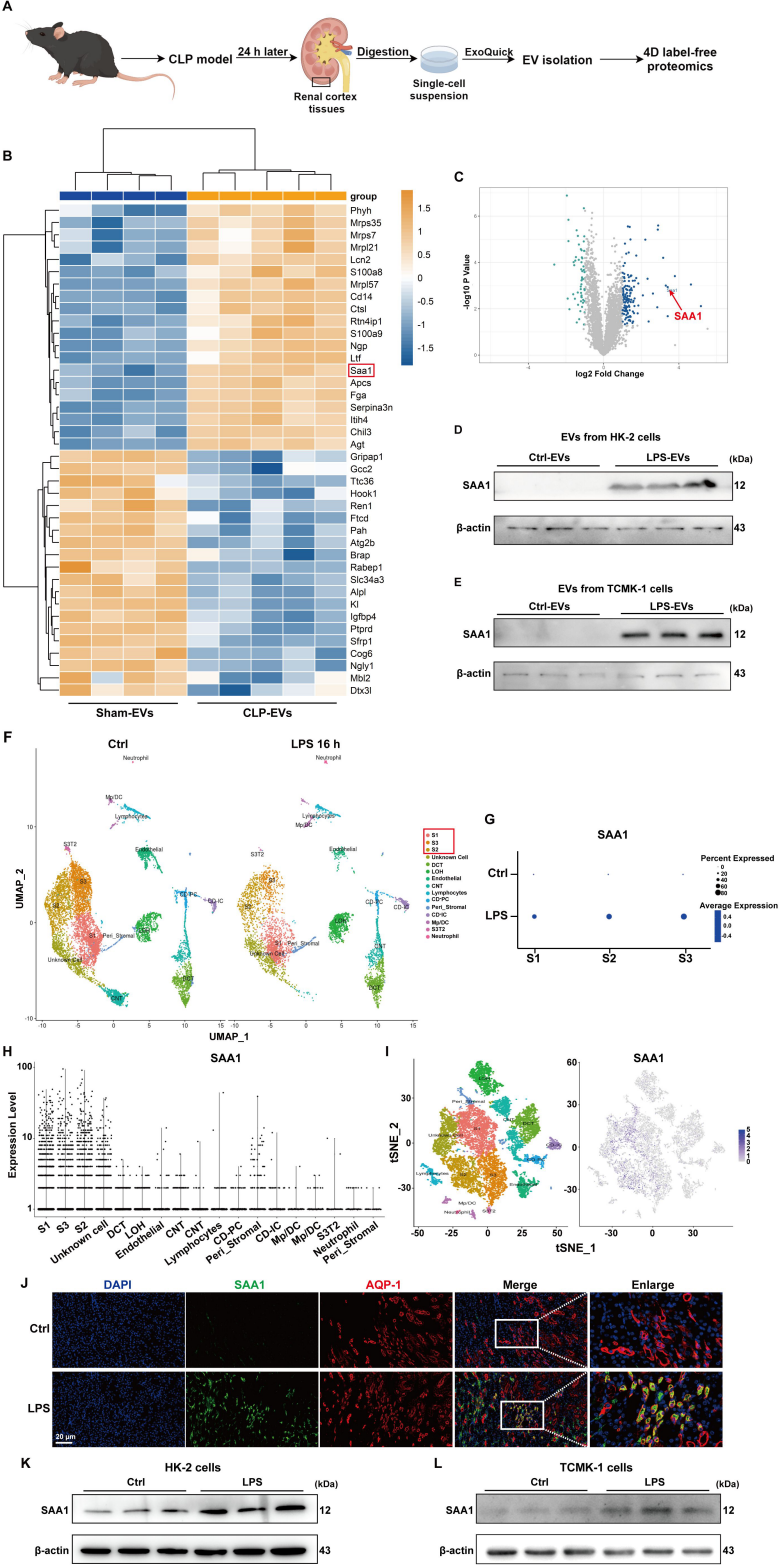
SAA1 was enriched in EVs secreted from LPS-stimulated TECs. **(A)** Schematic illustration of the experimental design. In brief, renal EVs were purified from the same weight of digested renal cortex tissues of the sham and CLP-treated mice via ExoQuick-TC reagent. Schematic was created with Figdraw.com. **(B)** Heatmap showing the top 20 proteins whose expression changed between the Sham-EVs and CLP-EVs (fold change > 2 or < 0.5). **(C)** Volcano plot displaying differentially expressed proteins between the Sham-EVs and CLP-EVs. **(D , E)** SAA1 expression in EVs isolated from the PBS/LPS-treated HK-2 and TCMK-1 cells was determined via western blotting. **(F)** UMAP plot showing clusters from the kidneys of the Ctrl and LPS groups. **(G)** Dot plot showing SAA1 expression across the three proximal tubule clusters (S1, S2, and S3) between the Ctrl and LPS groups. SAA1 gene expression in all clusters of LPS-treated kidneys was visualized via violin plots **(H)** and feature plots **(I, J)** Representative images of costaining for the proximal renal tubular cell marker AQP-1 (red) and SAA1 (green) in the kidneys of the Ctrl and LPS groups. Scale bar, 20 µm. **(K, L)** SAA1 expression in the PBS/LPS-treated HK-2 and TCMK-1 cells was determined via western blotting. S1, first segment of proximal tubule. S2, second segment of proximal tubule. S3, third segment of proximal tubule. DCT, distal convoluted tubule. LOH, Loop of Henle. CNT, connecting tubule. CD-PC, collecting duct-principle cells. Peri_Stromal, mixed pericyte and stromal cells. CD-IC, collecting duct-intercalated cells. Mp/DC, macrophage-dendritic cells. S3T2, S3 type 2 cells.

Next, we investigated the key molecules that mediate the effects of TECs-derived EVs on NETs formation. Given that proteins packaged in EVs are involved in regulating specific cellular functions under numerous physiological and pathological conditions, we performed 4D label-free proteomic analysis of EVs isolated from the kidney cortex of the sham and cecal ligation and puncture (CLP)-treated mice (as shown in **Figure 6A**). The isolated EVs were validated via transmission electron microscopy, NTA, and western blotting (**Supplementary Figure S3**). The top 20 changed proteins (fold change > 2 or < 0.5) are shown in **Figure 6B**. Among them, SAA1 was reported to induce neutrophilic airway inflammation by activating neutrophils along with NETs formation (14). SAA1 is shown in the volcano map (**Figure 6C**), and we further validated the enrichment of SAA1 in EVs isolated from the kidney cortex of LPS-treated mice (**Supplementary Figure S4**). EVs from LPS**-**stimulated HK-2 and TCMK-1 cells also contained more SAA1. (**Figures 6D, E**).

We also reanalyzed previously published scRNA-seq data of the kidney following LPS stimulation for 16 h. On the basis of the cluster-defining markers shown in previous work (24), the UMAP-based computational layout of epithelial clusters recapitulated the normal tubular segmental order of the nephron (**Figure 6F**). We next examined the differential gene expression between the control and LPS groups and found that all three segments (S1, S2, and S3) of the proximal tubules presented increased expression of SAA1 (**Figure 6G**). Violin plots and feature plots revealed that SAA1 was expressed mainly in the proximal tubule clusters of the LPS-stimulated mice (**Figures 6H, I**). As validated in our LPS-induced AKI model, the mRNA and protein levels of SAA1 in kidney tissue were significantly increased (**Supplementary Figure S5**). Double staining for SAA1 and AQP-1 (a marker for proximal renal tubular cells) further confirmed the significant upregulation of SAA1 in proximal tubules following LPS stimulation (**Figure 6J**). Western blot analysis also revealed markedly increased levels of SAA1 in LPS**-**stimulated HK-2 and TCMK-1 cells (**Figures 6K, L**). These results collectively suggest that LPS increases SAA1 expression in TECs, after which SAA1 is released extracellularly through EVs.

### EVs secreted from LPS-stimulated TECs promoted NETs formation and exacerbated AKI through SAA1

**Figure 7:**
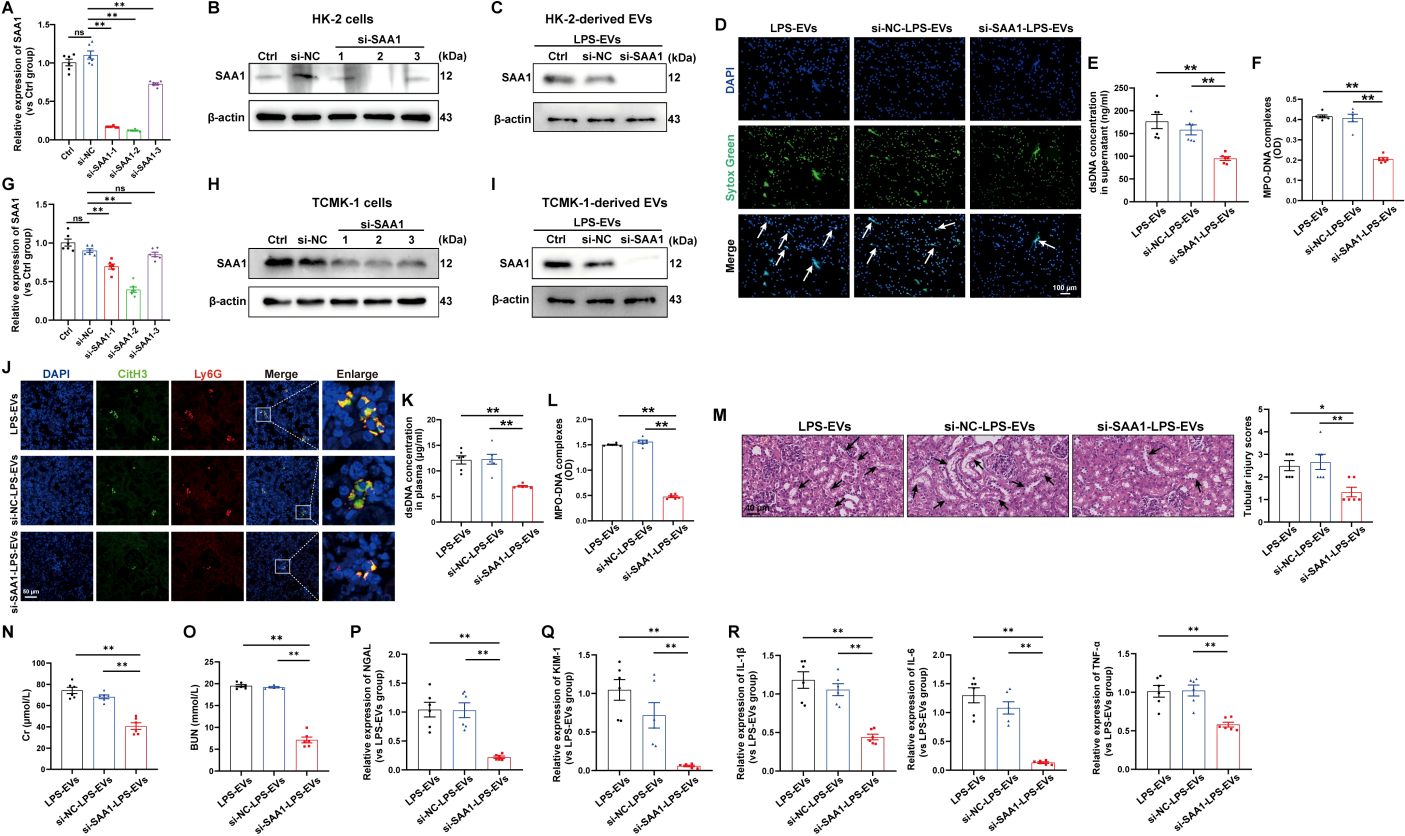
EVs secreted from LPS-stimulated TECs promoted NETs formation and exacerbated AKI through SAA1. SAA1-siRNAs were transfected into HK-2 cells before LPS stimulation. **(A, B)** At 24 h after LPS stimulation, SAA1 mRNA and protein expression in HK-2 cells was determined via RT–qPCR and western blotting. **(C)** SAA1 expression in EVs isolated from the siRNA-transfected HK-2 cells was assessed by western blotting. **(D)** Typical images of NETs formation were obtained via confocal microscopy via Sytox Green (green), where the white arrows indicate NETs. Scale bar, 100 μm. **(E, F)** Quantification of dsDNA and NETs components (MPO–DNA complexes) in the supernatants of cultured neutrophils via the PicoGreen assay and ELISA, respectively. **(G-R)** SAA1 siRNAs were transfected into TCMK-1 cells before LPS stimulation. **(G, H)** SAA1 mRNA and protein expression in TCMK-1 cells was determined 24 h after LPS stimulation. **(I)** SAA1 expression in EVs isolated from the siRNA-transfected TCMK-1 cells was assessed by western blotting. **(J)** Representative images showing the presence of NETs (Ly6G, red; citrullinated H3, green) in kidney tissues. Scale bar, 50 μm. **(K, L)** Quantification of dsDNA and MPO-DNA complexes in the plasma of the mice. **(M)** Representative images of H&E-stained kidneys (original magnification, 400×). Scale bar: 40 μm. The black arrows indicate tubule damage. Quantification of tubular injury via H&E staining. **(N, O)** Quantitation of Cr and BUN in blood samples from the mice in each group. **(P-R)** RT–qPCR analysis of NGAL, KIM-1, IL-1β, IL-6 and TNF-α mRNA levels in the kidney. One-way analysis of variance with Tukey’s multiple comparisons test was used for the analysis. The graphs present the means ± SEMs, n = 6; *P < 0.05, **P < 0.01 compared between two groups. ns, not significant.

To validate whether SAA1 packaged in EVs is responsible for NETs induction, we transfected SAA1-siRNAs into HK-2 cells before LPS stimulation, and SAA1 expression was effectively downregulated by si-SAA1-2 (**Figures 7A, B**). Afterward, EVs in the supernatant were isolated, and as shown by western blot, the expression of SAA1 was significantly reduced in EVs from the si-SAA1-2-transfected HK-2 cells (**Figure 7C**). After coculturing these EVs with neutrophils freshly isolated from healthy volunteers, we found that NETs formation was significantly inhibited in EVs from the SAA1 knockdown cell-treated group (**Figures 7D-F**).

Furthermore, we examined the effects of SAA1 packaged in TECs-derived EVs *in vivo*. TCMK-1 cells were also transfected with SAA1-siRNAs before LPS stimulation, and the mRNA and protein levels of SAA1 were efficiently decreased by si-SAA1-2 (**Figures 7G, H**). The expression of SAA1 in EVs from the si-SAA1-2-transfected cells was also significantly reduced (**Figure 7I**). Following the injection of these EVs, we observed significant inhibition of NETs formation both in kidney tissue and in plasma (**Figures 7J-L**) and alleviation of kidney injury and inflammation in EVs from the SAA1 knockdown cell-treated group (**Figures 7M-R**).

### SAA1 knockdown in TECs reduced NETs formation and alleviated LPS-induced AKI

**Figure 8:**
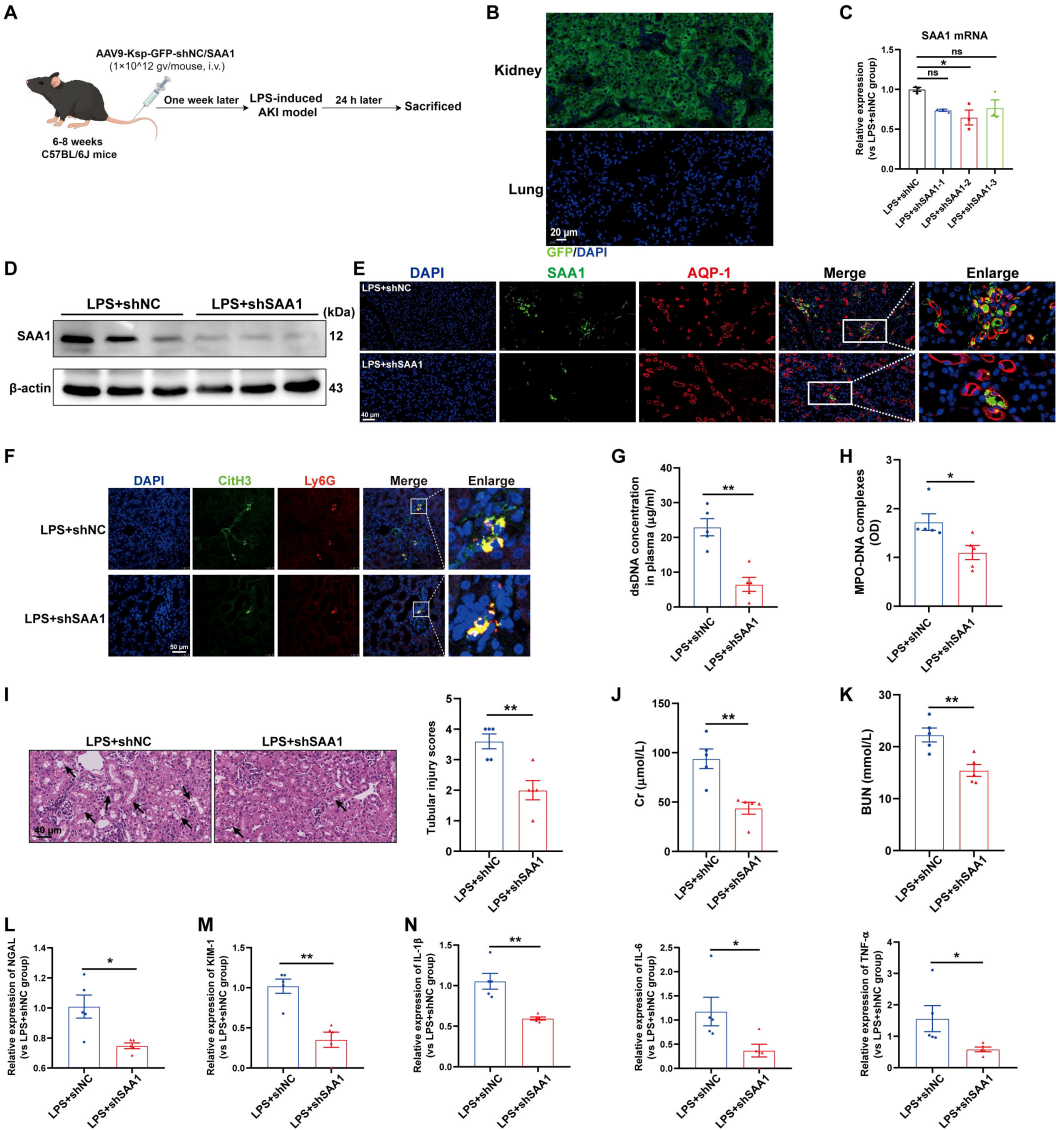
Knockdown of SAA1 in TECs reduced NETs formation and alleviated LPS-induced AKI. **(A)** Schematic diagram of the experimental design. Briefly, the mice were subjected to LPS stimulation at 7 days after AAV9-Ksp-GFP-shNC/SAA1 injection and were sacrificed at 24 h after LPS. Schematic was created with Figdraw.com. **(B)** Representative images of GFP fluorescence in the kidney and lung tissues of the AAV9-GFP-injected mice. Scale bar, 20 µm. **(C, D)** SAA1 mRNA and protein expression in kidney tissues was determined after the mice were treated with AAV9-shNC/SAA1. **(E)** Representative images of costaining for AQP-1 (red) and SAA1 (green) in the kidneys of the AAV9-shNC/SAA1-injected mice. Scale bar, 40 µm. **(F)** Representative images showing the presence of NETs (Ly6G, red; citrullinated H3, green) in kidney tissues. Nuclei were counterstained with DAPI (blue). Scale bar, 50 μm. **(G, H)** Quantification of dsDNA and MPO-DNA complexes in the plasma of the mice. **(I)** Representative images of H&E-stained kidneys (original magnification, 400×). Scale bars: 40 μm. The black arrows indicate tubule damage. Quantification of tubular injury via H&E staining. **(J, K)** Quantitation of Cr and BUN in blood samples from mice in each group. **(L-N)** RT–qPCR analysis of NGAL, KIM-1, IL-1β, IL-6 and TNF-α mRNA levels in the kidney. One-way analysis of variance with Tukey’s multiple comparisons test **(C)** or Student’s t test **(G-N)** was used for the analysis. The graphs represent the means ± SEMs, n = 5; *P < 0.05, **P < 0.01 compared between two groups. ns, not significant.

Next, we investigated whether the knockdown of SAA1 in TECs could reduce NETs formation and alleviate LPS-induced AKI *in vivo*. We generated an AAV9 vector carrying shRNAs against SAA1 and Ksp-cadherin, which was also used as the upstream promoter of SAA1 to specifically reduce SAA1 levels in TECs (**Figure 8A**). One week after injection, GFP fluorescence was clearly observed in nearly 100% of the tubular cells but was not detected in the lung tissue (**Figure 8B**). AAV9-shSAA1 significantly decreased the mRNA and protein levels of SAA1 in the kidney (**Figures 8C, D**), whereas did not affect SAA1 mRNA expression in the lung (**Supplementary Figure S6A**). Double-labeling of AQP-1 and SAA1 also revealed that SAA1 expression in TECs was lower in the AAV9-shSAA1 group than in the NC group (**Figure 8E**). As shown in **Figures 8F-H**, SAA1 knockdown in TECs significantly suppressed NETs formation at 24 h after LPS administration. Kidney histological lesions, plasma Cr and BUN levels, kidney injury markers and inflammatory factors in kidney tissues were all decreased in the AAV9-shSAA1 LPS-AKI mice (**Figures 8I-N**). These data indicated that the inhibition of SAA1 upregulation in TECs reduced NETs formation and alleviated LPS-induced AKI.

In addition, SAA1 knockdown in TECs decreased NETs formation in the lung and alleviated lung injury induced by LPS (**Supplementary Figures S6B-D**), indicating that, during sepsis, SAA1 released by TECs can mediate remote organ injury.

### SAA1 enriched in TECs-derived EVs promoted NETs formation via the TLR4/MAPK signaling pathway

**Figure 9:**
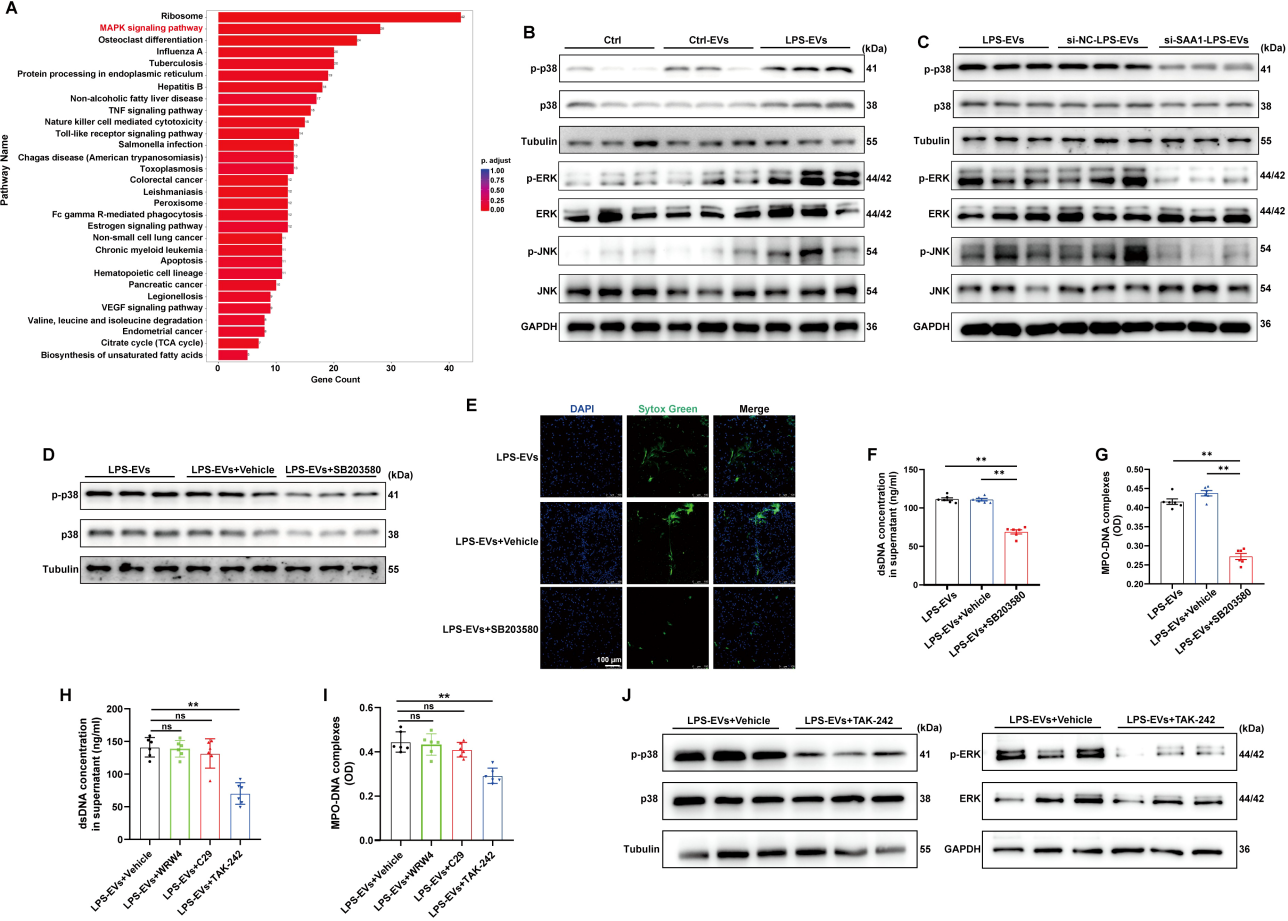
SAA1 enriched in TECs-derived EVs promoted NETs formation via the TLR4/MAPK signaling pathway. **(A)** Enrichment analysis of differentially expressed genes in neutrophils in the scRNA-seq data from the kidneys of the PBS/LPS-treated mice. **(B-D)** Representative western blot of MAPK pathway in neutrophils treated with PBS, Ctrl-EVs, LPS-EVs, and LPS-EVs from SAA1 knockdown HK-2 cells and LPS-EVs + SB203580 (a specific small molecule inhibitor of p38 MAPK phosphorylation, 30 µM). **(E)** Typical images of NETs formation were obtained via confocal microscopy via Sytox Green (green). Scale bar, 100 μm. **(F, G)** Quantification of dsDNA and NETs components (MPO–DNA complexes) in the supernatants of cultured neutrophils via the PicoGreen assay and ELISA, respectively. **(H-J)** WRW4 (10 µM), C29 (50 µM) or TAK-242 (50 µM) were added to the culture medium about 30 min before co-cultured with LPS-EVs. **(H, I)** Quantification of dsDNA and NETs components (MPO–DNA complexes) in the supernatants of cultured neutrophils via the PicoGreen assay and ELISA, respectively. **(J)** Representative western blot of MAPK pathway in neutrophils. One-way analysis of variance with Tukey’s multiple comparisons test was used for the analysis. The graphs present the means ± SEMs, n ≥ 3; **P < 0.01 compared between two groups. ns means “ns, not significant”.

To examine the signaling pathways that mediate NETs formation via SAA1 packaged in TECs-derived EVs, we reanalyzed the scRNA-seq data of the kidney as mentioned above (**Figure 6F**). Enrichment analysis of differentially expressed genes in neutrophils revealed significant enrichment of the MAPK signaling pathway (**Figure 9A**), indicating that LPS activates MAPK signaling pathways in neutrophils, which is consistent with a previously published study showing that activation of the MAPK pathway is required for NETs formation (25). Using western blot analysis, we also demonstrated that the phosphorylation levels of p38, JNK and ERK were increased following LPS-EVs treatment compared with that in the control group (**Figure 9B**), whereas EVs from SAA1 knockdown cells failed to increase p38, JNK and ERK phosphorylation compared with that in the NC group (**Figure 9C**). SB203580, a specific small molecule inhibitor of p38 MAPK phosphorylation (26), was sufficient to suppress p38 MAPK phosphorylation (**Figure 9D**) and NETs formation induced by LPS-EVs (**Figures 9E-G**).

Formyl peptide receptor 2 (FPR2) and Toll-like receptors (TLR2, 4) have been reported to be the receptors for SAA1 (27). The effects of LPS-EVs on NETs formation were significantly diminished after the cells were treated with the TLR4 antagonist TAK-242, while not the TLR2 antagonist C29 or the FPR2 inhibitor WRW4 (**Figures 9H, I**). Further, TLR4 antagonist also reversed the activation of MAPK pathway induced by LPS-EVs (**Figure 9J**). Taken together, our findings confirmed that SAA1 enriched in TECs-derived EVs promoted NETs formation via the TLR4/MAPK signaling pathway.

### Plasma TECs-derived EVs proportion and SAA1 level in plasma EVs were positively correlated with plasma NETs levels and poor outcomes in septic patients

**Figure 10:**
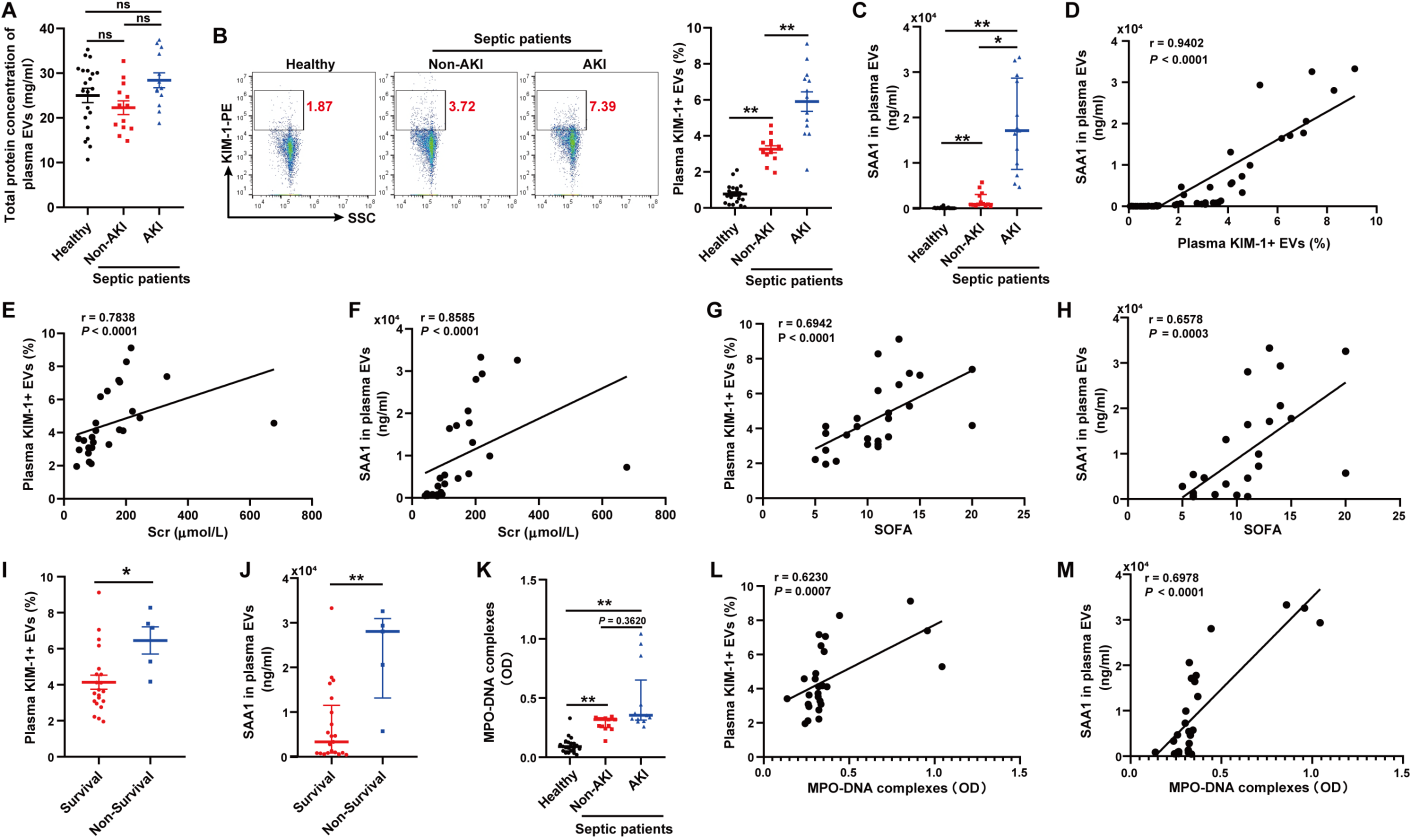
Plasma TECs-derived EVs proportion and SAA1 level in plasma EVs were positively correlated with plasma NETs levels and poor outcomes in septic patients. **(A)** Total protein concentration of EVs from equal plasma volumes of healthy controls (n = 21) and septic patients with or without AKI (n = 26). **(B)** Proportion of TECs-derived EVs identified in the plasma of healthy controls and septic patients with or without AKI as assessed by flow cytometry after gating by CD63-APC. **(C)** SAA1 concentration in EVs from equal plasma volumes as assessed via ELISAs. **(D)** Correlation of the SAA1 concentration in plasma EVs with the proportion of plasma TECs-derived EVs. **(E-H)** Association of the plasma TECs-derived EVs proportion or SAA1 concentration in plasma EVs with the Scr level and the SOFA score of septic patients. **(I, J)** The comparison of the plasma TECs-derived EVs proportion and SAA1 concentration in plasma EVs between the survival and non-survival septic patients. **(K)** Quantification of MPO-DNA complexes in the plasma of healthy controls and septic patients with or without AKI. **(L, M)** Correlation of the plasma TECs-derived EVs proportion and SAA1 concentration in plasma EVs with MPO-DNA complexes in plasma. Statistics: One-way analysis of variance with Tukey’s multiple comparisons test in **(A, B)**; the Kruskal–Wallis test followed by Dunn’s *post hoc* test in **(C, K)**; the Student’s t test in **(I)**; the Mann–Whitney U test in **(J)**; and the Spearman order correlation analysis in **(D-H, L, M)**. The data are presented as the means ± SEMs **(A, B, I)** or medians (25th–75th percentiles) **(C, J, K)**. *P < 0.05, **P < 0.01 compared between two groups. ns, not significant.

We isolated and purified plasma EVs from 21 healthy volunteers and 26 patients with sepsis. The septic patients were divided into two groups according to the occurrence of AKI (13 vs. 13). The total protein concentration of EVs among the three groups had no significant difference (**Figure 10A**). The proportion of EVs derived from TECs, identified by KIM-1, and the level of SAA1 in plasma EVs were higher in septic patients with AKI than those in healthy volunteers and septic patients without AKI as assessed by flow cytometry and ELISA respectively (**Figures 10B, C**). The proportion of TECs-derived EVs was positively related to the level of SAA1 in plasma EVs (**Figure 10D**). Correlation analysis revealed that plasma TECs-derived EVs proportion and SAA1 expression were positively correlated with the Scr level and the SOFA score in septic patients (**Figures 10E-H**). Plasma TECs-derived EVs proportion and SAA1 expression were also higher in the non-survivors among septic patients (**Figures 10I, J**). Furthermore, the plasma NETs concentration tended to increase in septic patients with AKI (**Figure 10K**) and was positively correlated with increased plasma TECs-derived EVs proportion and SAA1 expression in plasma EVs (**Figures 10L, M**). Additionally, we found that plasma TECs-derived EVs and SAA1 expression in plasma EVs were negative related to oxygenation index of septic patients (**Supplementary Figure S7**).

## Discussion

**Figure 11:**
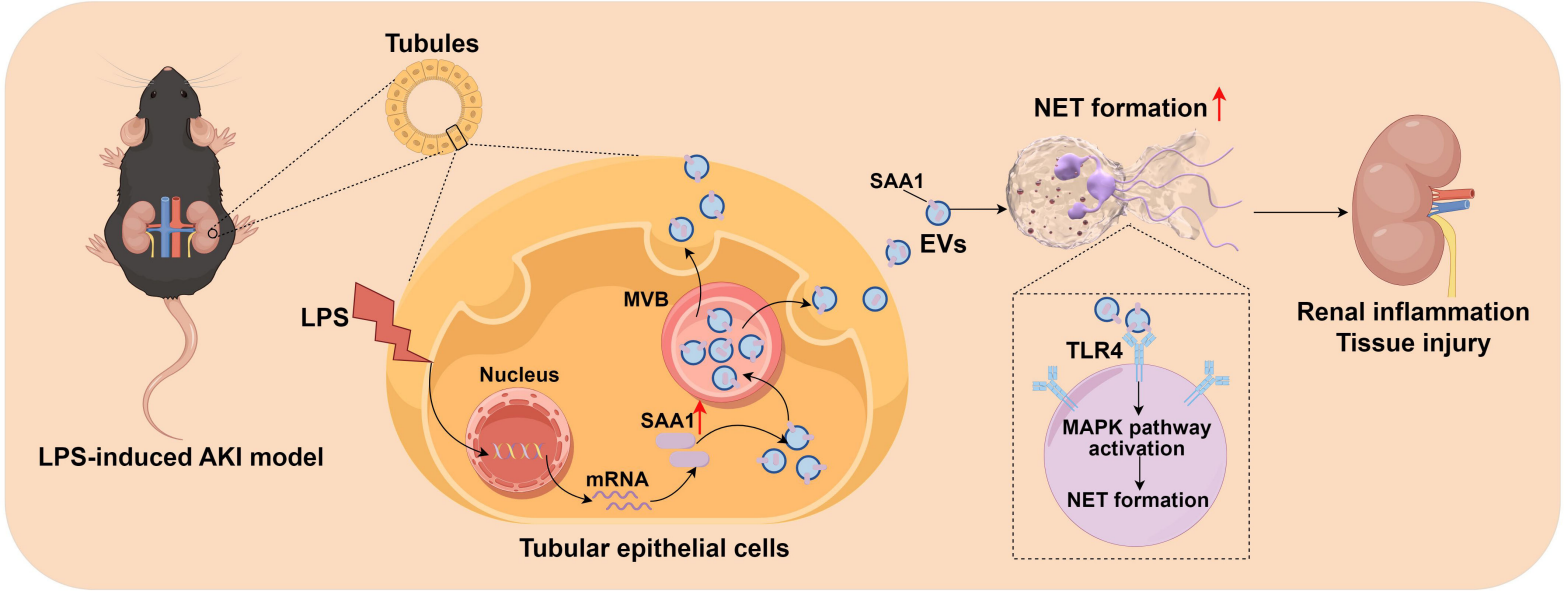
Schematic depiction of SAA1 delivered by tubular epithelial cell-derived extracellular vesicles exacerbates sepsis-associated acute kidney injury by promoting NETs formation. The graph was drawn on the Figdraw online website (Export ID: TIOORe3519).

In this study, we firstly demonstrated that EVs secreted from LPS-stimulated TECs aggravated AKI through promoting NETs formation. Combined proteomics and scRNA-seq analysis, we found that LPS increased SAA1 expression in TECs and then released extracellularly through EVs. Further mechanistic study showed that SAA1 packaged in TECs-derived EVs was responsible for NETs formation and AKI via activation of TLR4/MAPK signaling pathway in neutrophils (**Figure 11**). Specifically blocking EVs secretion from TECs or inhibiting SAA1 upregulation in TECs by AAV9s reduced NETs formation and alleviated LPS-induced AKI and remote lung injury. More importantly, we propose that the proportion of plasma TECs-derived EVs and SAA1 expression levels in EVs may serve as promising prognostic biomarkers for SA-AKI patients.

Neutrophils can expulse NETs to trap and kill pathogens, while excessive NETs formation can directly induce cytotoxic effects on tissues which, in turn, propagate inflammation and cause organ injury (28). Previous study has demonstrated that NETs induced tubular epithelial cell death in ischemia-reperfusion injury in the mouse kidney, and pretreatment with inhibitors of NETs formation reduced kidney injury (29). For SA-AKI, Ni et al., demonstrated that NETs are enriched in murine AKI of three distinct sepsis procedures, including CLP, LPS-induced endotoxemia and multidrug-resistant sepsis, and either pharmacological or genetic NETs interruption in combination with Fn14 blockade prolong mice survival and provide renal protection against sepsis (5). Consistent with their study, our study also showed that NETs formation was significantly enhanced in LPS-induced AKI, and NETs degrader DNase I could efficiently ameliorate kidney inflammation and tissue injury. Our study highlighted the significance of overzealous NETs formation in exacerbating sepsis and NETs can be an immune checkpoint for SA-AKI.

Increasing evidence has demonstrated that TECs-derived EVs play fundamental roles in the pathogenesis of kidney disease. Previous studies in both AKI and chronic kidney disease experimental models have shown that EVs-mediated TECs-neighbor cell crosstalk can cause the progression of tubulointerstitial inflammation and fibrosis (8, 10, 30). Here, we demonstrated that TECs exposed to LPS during SA-AKI communicated with neutrophils via EVs and led to NETs formation and kidney injury. Rab27a is known to be involved in the regulation of EVs secretion (31). As expected, the inhibition of EVs release from TECs via silence of Rab27a in TECs mitigated the LPS-induced NETs formation and AKI. These findings suggest a new role for TECs-derived EVs in aggravating SA-AKI by activating neutrophils.

Proteins can be sorted into EVs and selectively induce specific signals in recipient cells to modulate numerous processes (32). Our *in vitro* and *in vivo* experiments using siRNAs and AAVs revealed that SAA1-packaged in EVs released by LPS-stimulated TECs augmented the proinflammatory response during SA-AKI by inducing NETs formation. Aside by been regarded as a prognostic indicator for inflammatory diseases, SAA1 obtained multiple biological activities, including functioning as a pro-inflammatory molecular by attracting immune cells to the inflammation sites (33), and playing a promoting tumor progression role in liver and bladder cancers (34, 35). Those studies demonstrated that SAA1 mainly functions through unbound form to direct contact between cells, while our findings introduce a novel mode of action for SAA1 to be transported through EVs. A recent study also revealed that SAA stimulates renal dysfunction through promoting the IFN-iNOS-p38 MAPK axis (36), which was consistent with our study that SAA1 enriched in TECs-derived EVs promoted NETs formation via MAPK signaling pathway.

Interestingly, we demonstrated that AAV9-mediated blockade of TECs-EVs secretion or SAA1 upregulation attenuates LPS-induced lung injury. This suggests sepsis-injured TECs release SAA1-carrying EVs that disseminate via circulation, delivering pathogenic SAA1 to distant recipient cells. Our findings align with emerging consensus that EVs-facilitated interorgan communication contributes to septic kidney-lung axis derangement, offering insights into unified pathways of organ failure (37).

Recent studies have demonstrated a significant correlation between plasma EVs concentrations and the severity of organ dysfunction, as well as the prognostic mortality in critically ill septic patients (38). Consistently, we confirmed a significant increase of plasma TECs-derived EVs proportion and SAA1 concentration in plasma EVs, and positively correlated with poor prognosis and severity in septic patients. We further speculated that plasma TECs-derived EVs proportion and SAA1 concentration in plasma EVs may be attractive biomarkers for SA-AKI.

Our study had several limitations. First, TECs-derived EVs were isolated from the supernatant of *in vitro* culturing cells to evaluate their effects on NETs formation and AKI both *in vitro* and *in vivo*, not from the kidney cortex of septic mice using immunomagnetic beads or flow cytometry, due to their extreme fragility during the sorting process. Second, we only evaluated the effects of blocking EVs secretion from TECs or inhibiting SAA1 upregulation in TECs on AKI and remote lung injury following LPS challenge, while the other organs could also be influenced by TECs-derived EVs and thus need to be further addressed. Lastly, our clinical sample sizes are relatively small, larger clinical samples are needed to further validate the prognostic value of plasma TECs-derived EVs and SAA1 in plasma EVs for SA-AKI.

## Conclusion

This study revealed that TECs-derived EVs containing SAA1 exacerbated SA-AKI by promoting NETs formation through activation of the TLR4/MAPK pathway. TECs-derived EVs are also likely to play a role in kidney–lung crosstalk during sepsis. Additionally, plasma TECs-derived EVs proportion and SAA1 concentration in plasma EVs may be promising biomarkers for SA-AKI patients. These findings may add a new element to TECs–neutrophil crosstalk, and strategies to modify TECs-derived EVs and the cargo SAA1 could be a new avenue for developing therapeutics against SA-AKI.

## Data Availability

The proteomic data have been deposited to the ProteomeXchange Consortium via the iProX repository with the dataset identifier PXD055037. Any remaining information can be obtained from the corresponding author upon reasonable request.
