# Supplementary material for: Tubular epithelial cell-derived extracellular vesicles carrying serum amyloid A1 exacerbate sepsis-associated acute kidney injury by promoting NETs formation
Source: Front Immunol. 2025 Aug 27;16:1654295. doi: 10.3389/fimmu.2025.1654295 (PMC12420288; doi:10.3389/fimmu.2025.1654295)
Supplement: Supplementary file 1 [file Presentation1.pdf]

Supplementary Tables

Table S1 Demographic data of the included septic patients

**Characteristics**	**Septic patients without AKI (N = 13)**	**Septic patients with AKI (N = 13)**	***P* value**
Male sex, n (%)	10 (76.9)	4 (30.8)	**0.047**
Age, years	63 (58-72)	66 (61-72)	0.587
Mortality at 28 days, n (%)	1 (7.7)	4 (30.8)	0.322
Comorbidities, n (%)			
Arterial hypertension	4 (30.8)	5 (38.5)	1.000
Diabetes mellitus	3 (23.1)	1 (7.7)	0.593
Source of sepsis, n (%)			
Urinary	3 (23.1)	2 (15.4)	1.000
Abdominal	8 (61.5)	9 (69.2)	1.000
Others	2 (15.4)	2 (15.4)	1.000
Hemodynamic data			
Heart rate/min	89.6 ± 23.2	95.2 ± 30.4	0.601
Mean arterial pressure, mmHg	82.7 (80-90)	73 (72-88)	0.304
Norepinephrine dosage, µg/kg/min	0.03 (0.02-0.05)	0.1 (0.05-1)	**0.008**
Ventilatory data			
Respirate rate/min	12 (12-15)	12 (12-25)	0.336
PaCO2 (mmHg)	38.4 ± 7.8	35.3 ± 14.1	0.505
PaO2/FIO2 (Kpa)	300.6 ± 110.3	258.1 ± 131.7	0.381
Use of mechanical ventilation, n (%)	11 (84.6)	11 (84.6)	1.000
Hematologic and inflammatory data			
Neutrophils, 10^9/L	11.4 (9.7-17.7)	21.1 (13.7-25.7)	**0.017**
Hemoglobin, g/dL	93.4 ± 17.0	88.2 ± 25.5	0.550
Platelets, 10^9/L	163 (101-188)	103 (65-152)	0.057
Scr, µmol/L	81.4 (64-95)	190.8 (140-221)	**< 0.0001**
Lactate, mmol/L	1.2 (1.1-1.4)	3.2 (1.8-8.4)	**0.003**
CRP, mg/dL	184.6 (124.8-226.3)	228.6 (198.3-293.8)	0.113
Procalcitonin, ng/mL	1.9 (0.5-5.4)	47.8 (15.1-94.5)	**< 0.0001**
SOFA score	9.3 ± 3.9	12.1 ± 3.6	0.070

Scr: Serum creatinine, CRP: C-reactive protein, SOFA: Sequential Organ Failure Assessment.

Data are expressed as number (%), mean ± SD, or median (25th-75th percentile). Fisher's exact test, Student’s t test or Mann-Whitney U test were used for the analysis.

Table S2 Primer sequences

Gene symbol	Forward Primer (5'-3')	Reverse Primer (5'-3')
IL-1β	TGCCACCTTTTGACAGTGATG	AAGGTCCACGGGAAAGACAC
IL-6	AAAGAGTTGTGCAATGGCAATTCT	AAGTGCATCATCGTTGTTCATACA
TNF-α	CATCTTCTCAAAATTCGAGTGACAA	TGGGAGTAGACAAGGTACAACCC
KIM-1	ACATATCGTGGAATCACAACGAC	ACTGCTCTTCTGATAGGTGACA
NGAL	GCAGGTGGTACGTTGTGGG	CTCTTGTAGCTCATAGATGGTGC
Rab27a	AAGGGATAGAGCACAGCGAG	AACTCTGTGCCTCACCTCAA
Human-SAA1	CTGCAGAAGTGATCAGCG	ATTGTGTACCCTCTCCCC
Mouse-SAA1	GGATGAAGCTACTCACCAGCC	TCCCCAGCCCCTTGGAAA
GAPDH	GCATGGCCTTCCGTGTTC	GATGTCATCATACTTGGCAGGTTT

Supplementary Results

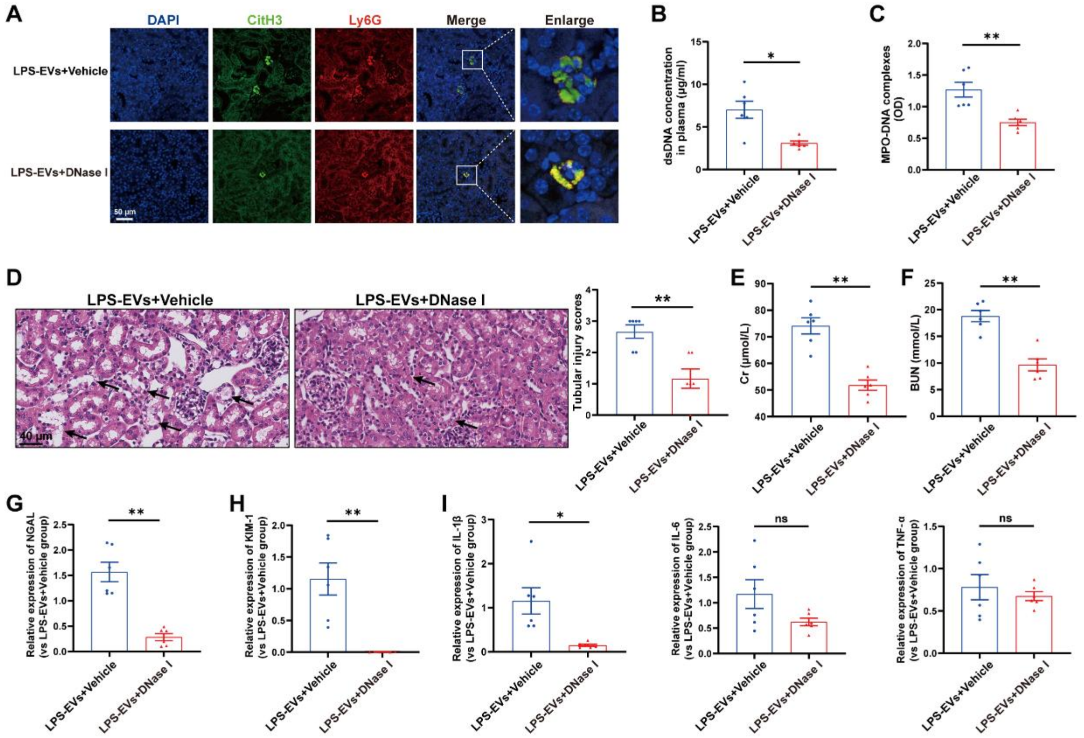

Figure S1 EVs secreted from LPS-stimulated tubular epithelial cells aggravated AKI through promoting NET formation. DNase I (5 mg/kg) was given with an *i.p.* injection 30 min after LPS-EVs administration, and equal volume of normal saline was used as vehicle. (**A**) Representative images showing the presence of NETs (Ly6G, red; citrullinated H3, green) in the kidney tissues. Nuclei were counterstained with DAPI (blue). Scale bar, 50 μm. (**B** and **C**) Quantification of dsDNA and circulating NET structures (MPO-DNA complexes) in the plasma of mice using PicoGreen fluorescent quantification and ELISA, respectively. (**D**) Representative images of H&E-stained kidneys (original magnification, ×400). Scale bars: 40 μm. Black arrows indicate tubule damage. Quantification of tubular injury based on H&E staining. (**E** and **F**) Quantitation of Cr and BUN in blood samples from mice per group. (**G-I**) RT-qPCR analysis of NGAL, KIM-1, IL-1β, IL-6 and TNF-α mRNA levels in kidneys. Student's t test was used for the analysis. Graphs represent means ± SEM, n = 6; **P* < 0.05, ***P* < 0.01 compared within two groups. ns, not significant.

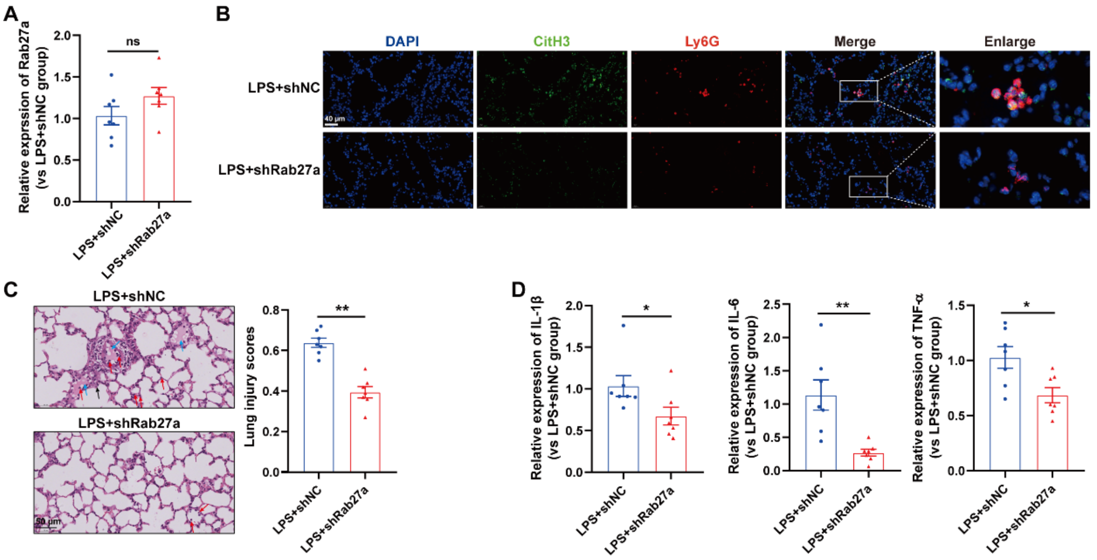

Figure S2 Blocking EV secretion from tubular epithelial cells reduced NET formation and alleviated LPS-induced lung injury. (**A**) RT-qPCR analysis of Rab27a mRNA in the lung tissues. (**B**) Representative images showing the presence of NETs (Ly6G, red; citrullinated H3, green) in the lung tissues. Nuclei were counterstained with DAPI (blue). Scale bar, 40 μm. (**C**) Evaluation of lung histology by H&E staining (magnifcation×400). Red arrows indicate neutrophils in the alveolar and interstitial space, blue arrows indicate proteinaceous debris filling, and black arrows indicate thickening of the alveolar walls. Scale bar, 50 μm. Lung injury scores were assessed. (**D**) Detection of inflammatory cytokine mRNA (IL-1β, IL-6, TNF-α) expression in lung tissues by RT-qPCR. Student's t test was used for the analysis. Graphs represent means ± SEM, n = 7; **P* < 0.05, ***P* < 0.01 compared within two groups. ns, not significant.

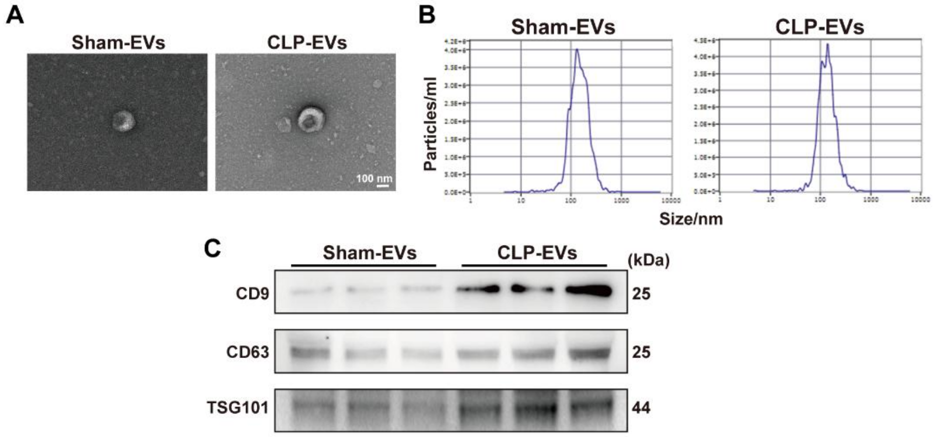

Figure S3 Characterization of the EVs isolated from the digested renal cortex tissues of Sham and CLP mice. (**A**) Electron micrograph of EVs isolated from the same weight of digested renal cortex tissues of Sham and CLP mice. Scale bar, 100 nm. (**B**) EVs size distribution was measured by NanoSight tracking analysis. (**C**) CD9, CD63 and TSG101 protein expression in EVs were quantified by Western blot loaded with equal amounts of EVs protein (20 μg).

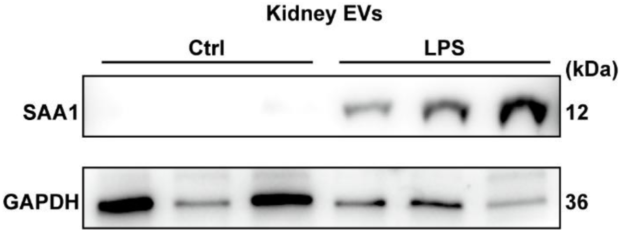

Figure S4 WT C57BL/6 mice were administered with PBS/LPS (10 mg/kg of body weight) intraperitoneally. After 24 h, renal EVs were purified from the same weight of digested renal cortex tissues via ExoQuick-TC reagent. The expression of SAA1 in EVs was determined by western blot.

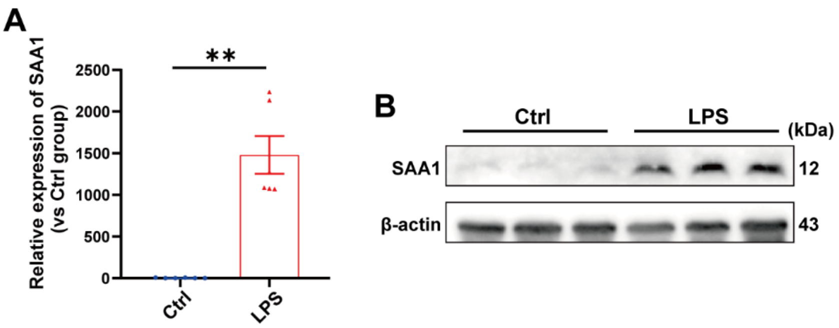

Figure S5 LPS increased SAA1 expression in the kidney. WT C57BL/6 mice were administered with LPS (10 mg/kg of body weight) intraperitoneally. After 24 h, kidneys were harvested and mRNA and protein levels of SAA1 in kidney tissues were detected using RT-qPCR and western blot. Student's t test was used for the analysis. Graphs represent means ± SEM, n = 6; ***P* < 0.01 compared within two groups.

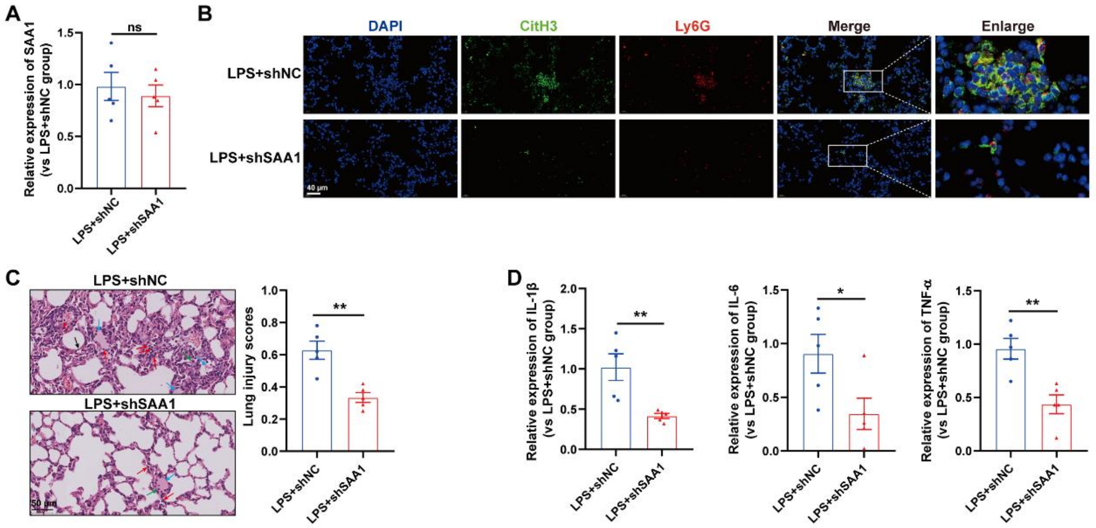

Figure S6 Knockdown of SAA1 in tubular epithelial cells reduced NET formation and alleviated LPS-induced lung injury. (**A**) RT-qPCR analysis of SAA1 mRNA in the lung tissues. (**B**) Representative images showing the presence of NETs (Ly6G, red; citrullinated H3,

green) in the lung tissues. Nuclei were counterstained with DAPI (blue). Scale bar, 40 μm. (**C**) Evaluation of lung histology by H&E staining (magnifcation×400). Red arrows indicate neutrophils in the alveolar and interstitial space, green arrows indicate alveolar macrophages, blue arrows indicate proteinaceous debris filling, and black arrows indicate thickening of the alveolar walls. Scale bar, 50 μm. Lung injury scores were assessed. (**D**) Detection of inflammatory cytokine mRNA (IL-1β, IL-6, TNF-α) expression in lung tissues by RT-qPCR. Student's t test was used for the analysis. Graphs represent means ± SEM, n = 5; **P* < 0.05, ***P* < 0.01 compared within two groups. ns, not significant.

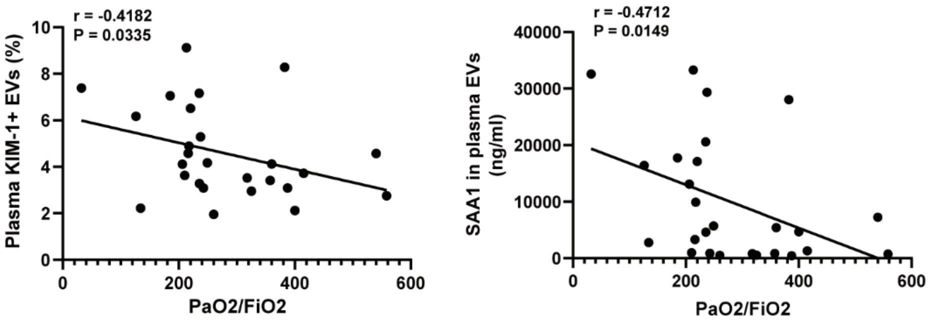

Figure S7 Correlation of the plasma TEC-derived EV proportion and SAA1 concentration in plasma EVs with PaO2/FiO2 of septic patients. Spearman order correlation analysis was used in the analysis.
