# Supplementary material for: Tubular epithelial cell-derived extracellular vesicles carrying serum amyloid A1 exacerbate sepsis-associated acute kidney injury by promoting NETs formation
Source: Front Immunol. 2025 Aug 27;16:1654295. doi: 10.3389/fimmu.2025.1654295 (PMC12420288; doi:10.3389/fimmu.2025.1654295)
Supplement: Supplementary file 2 [file Presentation2.pdf]

Supplementary Methods

Renal function analysis

Plasma Cr and BUN levels were measured based on a commercial assay kit (Jiancheng, Nanjing, China) according to the manufacturer's instructions.

Renal histological assessment and immunostaining

Kidney tissues were fixed, embedded in paraffin, and sectioned into 4 μm sections. To analyze pathological changes, kidney sections were stained with hematoxylin and eosin (H & E). Tubular injury was scored based on the percentage of injured area. Injury was semiquantitatively scored as follows: 0, no damage; 1, <25%; 2, 25 to ~50%; 3, 50 to ~75%; 4, >75%. An average percentage was calculated by 10 randomly chosen fields (×400) from each mouse.

For immunofluorescence detection, paraffin-embedded kidney tissues were sectioned, blocked with PBS containing 10% goat serum, and permeabilized with PBS/0.1% Triton X-100. The slides were incubated with antibodies against Ly6G (#sc53515, Sigma, 1:100), citrullinated histone H3 (CitH3) (#ab5103, Abcam, 1:200), AQP-1 (#ab168387, Abcam, 1:200), SAA1 (#A14553, ABclonal, 1:200) and CD63 (#YT5525, Immunoway, 1:100) respectively. Following incubation with the primary antibody, the slides were washed with PBS, incubated for 1 h with species-specific secondary antibody coupled with Alexa Fluor Dye, counterstained with DAPI, and finally viewed under a confocal microscope.

EV characterization

The morphology of the EVs was characterized using transmission electron microscopy. The size distribution of EVs was assessed by NanoSight tracking analysis. The surface markers of EVs (CD9, CD63 and TSG101) were identified by western blot, loading with the same amount of EV protein (20 μg).

Transmission electron microscopy

After isolation of EVs, the pellets were fixed with 2% glutaraldehyde in 0.1 M phosphate buffer

(pH 7.4). The fixed pellets were placed on 100-mesh, carbon-coated, formvar-coated nickel grids treated with poly-L-lysine for 30 min. After washing the samples with several drops of PBS, samples were incubated with drops of buffered 1% glutaraldehyde for 5 min and then washed several times with drops of distilled water. Afterward, samples were negatively stained with drops of Millipore-filtered aqueous 4% uranyl acetate for 5 min. Stain was blotted dry from the grids with filter paper, and samples were allowed to dry. The microscopy images were captured by a JEOL JEM-1400 transmission electron microscope operating at 120 kV.

EVs uptake studies

EVs purified from TECs were stained in the presence of 10 μM highly lipophilic nature of dyes Dil (#C1036, Beyotime Biotechnology, Shanghai, China) for 30 minutes at 37°C. Then the EVs were washed with PBS and isolated using ExoQuick-TC to remove remaining dyes. Labelled EVs were resuspended in 200 μl of PBS and then co-cultured with freshly isolated neutrophils for 3 h at 37°C. Then, confocal microscopy and flow cytometry were used to detect EVs uptake efficiency.

Flow cytometry detection of EVs

100 μl EVs were incubated with 10 μl aldehyde/sulfate latex beads (4 mm diameter; Thermo Fisher Scientific, USA) for 15 min at 4°C. PBS was then added to the EVs to reach a total volume of 400 μl, followed by incubation at 4°C with gentle agitation overnight and centrifuged at 1000 g for 5 min. To determine the proportion of TEC-derived EVs in the whole EV quantity isolated from plasma, EV-coated beads were stained with PE anti-human KIM-1 (#353904, Biolegend, USA), APC anti-human CD63 antibody (#353008, Biolegend, USA), or human IgG for 30 min at room temperature. Then, 1 ml PBS was added to wash beads twice and resuspended in 300 μl PBS. EV-coated beads were gated on FSC and SSC, and analyzed by flow cytometry.

NET quantification assay

To quantify NETs in the cell culture supernatant and plasma, we used the PicoGreen dsDNA Quantification Kit (Invitrogen, Carlsbad, CA, USA) and a capture ELISA based on

myeloperoxidase (MPO) associated with DNA[2]. For ELISA analysis of NET concentration, 1 μg/mL anti-MPO mAb was used as a capture antibody with Cell Death Detection ELISA (Roche, Indianapolis, IN, USA) according to the instructions.

NET formation was also visualized by confocal microscopy. Neutrophils were allowed to settle on glass coverslips precoated with poly-l-lysine (#ST509, Beyotime Biotechnology, Shanghai, China) for 30 min prior to being treated for a specific period of time. Neutrophils were incubated with 1 μM SYTOX Green reagent (#S7020, Invitrogen, USA) at 37 °C for 15 min. Nuclei were counterstained using DAPI, and the cells were mounted in Antifade Mounting Medium (#P0126, Beyotime Biotechnology, Shanghai, China) for imaging with a confocal microscope. For each slice, 5 random fields were captured and analysed.

4D label-free proteomics analysis

EVs isolated from the kidney cortex of sham and CLP mice were subjected to 4D label-free proteomics analysis. Briefly, proteins were extracted, quantified, separated via SDS-PAGE, and enzymatically digested. A timsTOF Pro mass spectrometer (Bruker, Bremen, Germany) was subsequently used to obtain the raw data. The raw data were analyzed using MaxQuant software version 1.6.17.0. The cutoff of global false discovery rate (FDR) for peptide and protein identification was set to 0.01. Protein abundance was calculated on the basis of the normalized spectral protein intensity (LFQ intensity). Proteins which fold change > 2 or < 0.5 and *P* value (Student's t test) < 0.05 were considered to be a differentially expressed protein. Visualization of the result was generated using functions from ggplot2 R package (version 3.3.3).

Single-cell RNA-sequencing analysis

We analysed single-cell RNA-sequencing (scRNA-seq) datasets (GEO GSE151658) of kidney in a murine endotoxemia model which was published previously[3]. Mice were subjected to a single dose of 5 mg/kg LPS via tail vein injection and sacrificed at 0 and 16 h after LPS. Seurat R package (version 3.2.3) was used for quality control and normalized steps. Batch effects were corrected using the Harmony R package (version 0.1.0). Subsequently, datasets were clustered by the Seurat FindNeighbors and FindClusters functions. Then, Uniform Manifold

Approximation and Projection (UMAP) was used to perform dimensional reduction and cluster-defining markers published in the previous work[3] were used for cluster annotation.

Quantitative real-time PCR

The mRNA expression levels of KIM-1, NGAL, IL-1β, IL-6, TNF-α, Rab27a and SAA1 were analysed via HiScript III SuperMix (#R323, Vazyme) and Universal SYBR qPCR Master Mix kits (#027E2201CB, Vazyme), with GAPDH as the internal reference gene. The target genes were quantified in a real-time fluorescence-based quantitative PCR machine (QuantStudio™ 5, Thermo Fisher). The sequences of the primers used in this study are listed in (**Table S2**). The relative gene expression was quantified by the $2^{-\triangle\triangle CT}$ method.

Western blot analysis

EVs, cells and tissues were lysed in ice-cold RIPA Lysis buffer supplemented with protease inhibitor cocktail, sonicated, and the protein concentration was determined by BCA assay. Lysates were then boiled in SDS sample buffer and resolved on 10-12% SDS-PAGE gel. Immunoblots were incubated overnight with primary antibodies against CD9 (#A19027; ABclonal, Wuhan, China), CD63 (#A5271; ABclonal), TSG101 (#A1692; ABclonal), SAA1 Polyclonal antibody (#16721-1-AP; Proteintech, for detection of SAA1 in EVs), SAA1 (#A14553; ABclonal, for detection of SAA1 in TCMK-1 cells), SAA1 (#ab207445; Abcam, for detection of SAA1 in HK-2 cells), Rab27a (#A23993; ABclonal), p38 (#14064-1-AP; Proteintech), p-p38 (#4511; Cell Signaling Technology), ERK (#4695; Cell Signaling Technology), p-ERK (#4370; Cell Signaling Technology), JNK (#AF1048; Beyotime), p-JNK (#AF1762; Beyotime), β-actin (#66009-1; Proteintech), Tubulin (#14555-1-AP; Proteintech). Immunoblots were examined using an ECL detection reagent (Vazyme, Nanjing, China).

References

1. Tonghui X, Jialin G, Maozeng W, Jiali W, Kehui Y, Chang P, Jiaojiao P, Li X, Qiuhuan Y, Mengyang X et al: Aldehyde dehydrogenase 2 protects against acute kidney injury by regulating autophagy via the Beclin-1 pathway. JCI Insight 2021, 6(15).
2. Jiao Y, Li W, Wang W, Tong X, Xia R, Fan J, Du J, Zhang C, Shi X: Platelet-derived exosomes promote neutrophil extracellular trap formation during septic shock. Critical

care (London, England) 2020, 24(1):380.
3. Danielle J, Jered M, Thomas W M, Amy Z, Farooq S, Xiaoling X, Hongyu G, Yun-Long L, Kimberly S C, Ying-Hua C et al: The orchestrated cellular and molecular responses of the kidney to endotoxin define a precise sepsis timeline. Elife 2021, 10(0).
